# Exploring Natural Product Activity and Species Source Candidates for Hunting ABCB1 Transporter Inhibitors: An In Silico Drug Discovery Study

**DOI:** 10.3390/molecules27103104

**Published:** 2022-05-12

**Authors:** Mahmoud A. A. Ibrahim, Khlood A. A. Abdeljawaad, Alaa H. M. Abdelrahman, Laila A. Jaragh-Alhadad, Hesham Farouk Oraby, Eslam B. Elkaeed, Gamal A. H. Mekhemer, Gamal A. Gabr, Ahmed M. Shawky, Peter A. Sidhom, Mahmoud E. S. Soliman, Mahmoud F. Moustafa, Paul W. Paré, Mohamed-Elamir F. Hegazy

**Affiliations:** 1 Computational Chemistry Laboratory, Chemistry Department, Faculty of Science, Minia University, Minia 61519, Egypt; kh.abdeljawaad@compchem.net (K.A.A.A.); a.abdelrahman@compchem.net (A.H.M.A.); gmekhemer@mu.edu.eg (G.A.H.M.); 2 Department of Chemistry, Faculty of Science, Kuwait University, Safat 13060, Kuwait; laila.alhadad@ku.edu.kw; 3 Deanship of Scientific Research, Umm Al-Qura University, Makkah 21955, Saudi Arabia; hforaby@uqu.edu.sa; 4 Department of Crop Science, Faculty of Agriculture, Zagazig University, Zagazig 44519, Egypt; 5 Department of Pharmaceutical Sciences, College of Pharmacy, AlMaarefa University, Riyadh 13713, Saudi Arabia; ikaeed@mcst.edu.sa; 6 Department of Pharmacology and Toxicology, College of Pharmacy, Prince Sattam Bin Abdulaziz University, Al-Kharj 11942, Saudi Arabia; g.gabr@psau.edu.sa; 7 Agricultural Genetic Engineering Research Institute (AGERI), Agricultural Research Center, Giza 12619, Egypt; 8 Science and Technology Unit (STU), Umm Al-Qura University, Makkah 21955, Saudi Arabia; amesmail@uqu.edu.sa; 9 Department of Pharmaceutical Chemistry, Faculty of Pharmacy, Tanta University, Tanta 31527, Egypt; peter.ayoub@pharm.tanta.edu.eg; 10 Molecular Bio-Computation and Drug Design Research Group, School of Health Sciences, University of KwaZulu-Natal, Westville, Durban 4000, South Africa; soliman@ukzn.ac.za; 11 Department of Biology, College of Science, King Khalid University, Abha 9004, Saudi Arabia; mfmostfa@kku.edu.sa; 12 Department of Botany and Microbiology, Faculty of Science, South Valley University, Qena 83523, Egypt; 13 Department of Chemistry & Biochemistry, Texas Tech University, Lubbock, TX 79409, USA; paul.pare@ttu.edu; 14 Chemistry of Medicinal Plants Department, National Research Centre, 33 El-Bohouth St., Dokki, Giza 12622, Egypt

**Keywords:** ABCB1, multidrug resistance (MDR), NPASS, molecular docking, molecular dynamics (MD) simulations

## Abstract

The P-glycoprotein (P-gp/ABCB1) is responsible for a xenobiotic efflux pump that shackles intracellular drug accumulation. Additionally, it is included in the dud of considerable antiviral and anticancer chemotherapies because of the multidrug resistance (MDR) phenomenon. In the search for prospective anticancer drugs that inhibit the ABCB1 transporter, the Natural Product Activity and Species Source (NPASS) database, containing >35,000 molecules, was explored for identifying ABCB1 inhibitors. The performance of AutoDock4.2.6 software to anticipate ABCB1 docking score and pose was first assessed according to available experimental data. The docking scores of the NPASS molecules were predicted against the ABCB1 transporter. Molecular dynamics (MD) simulations were conducted for molecules with docking scores lower than taxol, a reference inhibitor, pursued by molecular mechanics-generalized Born surface area (MM-GBSA) binding energy estimations. On the basis of MM-GBSA calculations, five compounds revealed promising binding affinities as ABCB1 inhibitors with Δ*G*_binding_ < −105.0 kcal/mol. The binding affinity and stability of the identified inhibitors were compared to the chemotherapeutic agent. Structural and energetical analyses unveiled great steadiness of the investigated inhibitors within the ABCB1 active site throughout 100 ns MD simulations. Conclusively, these findings point out that NPC104372, NPC475164, NPC2313, NPC197736, and NPC477344 hold guarantees as potential ABCB1 drug candidates and warrant further in vitro/in vivo tests.

## 1. Introduction

P-glycoprotein (P-gp) is a member of the ATP-binding cassette (ABC) superfamily of transporters. P-gp encoded via the ABCB1/MDR1 gene is a multidrug transporter that has a critical role in safeguarding tissues from hazardous chemicals in humans by pumping the xenobiotics outside the cells [1,2,3]. This is relevant from a pharmacological standpoint because it lowers the absorption of some orally delivered medicines and limits therapeutic delivery across the blood-brain barrier, where the ABCB1 is extremely expressed [4]. ABCB1 has a considerable role in drug absorption, distribution, metabolism, and excretion (ADME) in the human body [5]. In some malignancies, the ABCB1 is overexpressed and its ability to extrude a variety of chemicals using its efflux pumps contributes to multidrug resistance (MDR) [6]. As the impact of ABCB1 in MDR has become evident, researchers have been focusing their efforts on discovering potent ABCB1 inhibitors that can conquer MDR [6,7]. Various inhibitory drugs were designed towards the ABCB1 transporter and exhibited good efficacy in cellular experiments; but, due to the poor selectivity, inadequate efficacy, or high toxicity, the majority of those drugs failed in clinical trials [8]. Consequently, there is still a demand to discover novel ABCB1 inhibitors to overcome MDR in tumor cells.

Nature has contributed to modern drug discovery [9,10,11]; approximately 40% of Food and Drug Administration (FDA)-approved drugs are originated from either natural products (NPs) or their derivatives. As a result, NPs have gotten much attention from researchers who are looking for prospective therapeutics to treat several diseases like human immunodeficiency virus (HIV) [12], malaria [13], and neoplastic disease [14]. Because of the long-developing time and expensive cost of extracting and detecting NPs, researchers built chemical libraries containing molecules from natural sources [15,16]. Natural Product Activity and Species Source (NPASS) database is one of the most popular natural products databases comprising 35,032 NPs. This study was undertaken to screen the NPASS database for prospective drug candidates that could inhibit the ABCB1 binding pocket and block its efflux pump function. Molecular docking computations were conducted in three stages for the sake of filtering the most potent NPASS compounds against the ABCB1 transporter. The NPASS compounds with the lowest docking scores were subjected to molecular dynamics (MD) simulations. Furthermore, the molecular mechanics-generalized Born surface area (MM-GBSA) method was utilized to compute the inhibitor-ABCB1 binding affinities throughout the time of the simulation. The structural and energetical stabilities of the top potent NPASS compounds bound with the ABCB1 transporter were then investigated throughout the 100 ns MD course. These findings shed light on the prospectivity of NPASS compounds as potential drug candidates to combat MDR and thus symbolize an effective factor for rational discovery of modulators of other receptors.

## 2. Results and Discussion

### 2.1. Assessment of In Silico protocol

**Figure 1:**
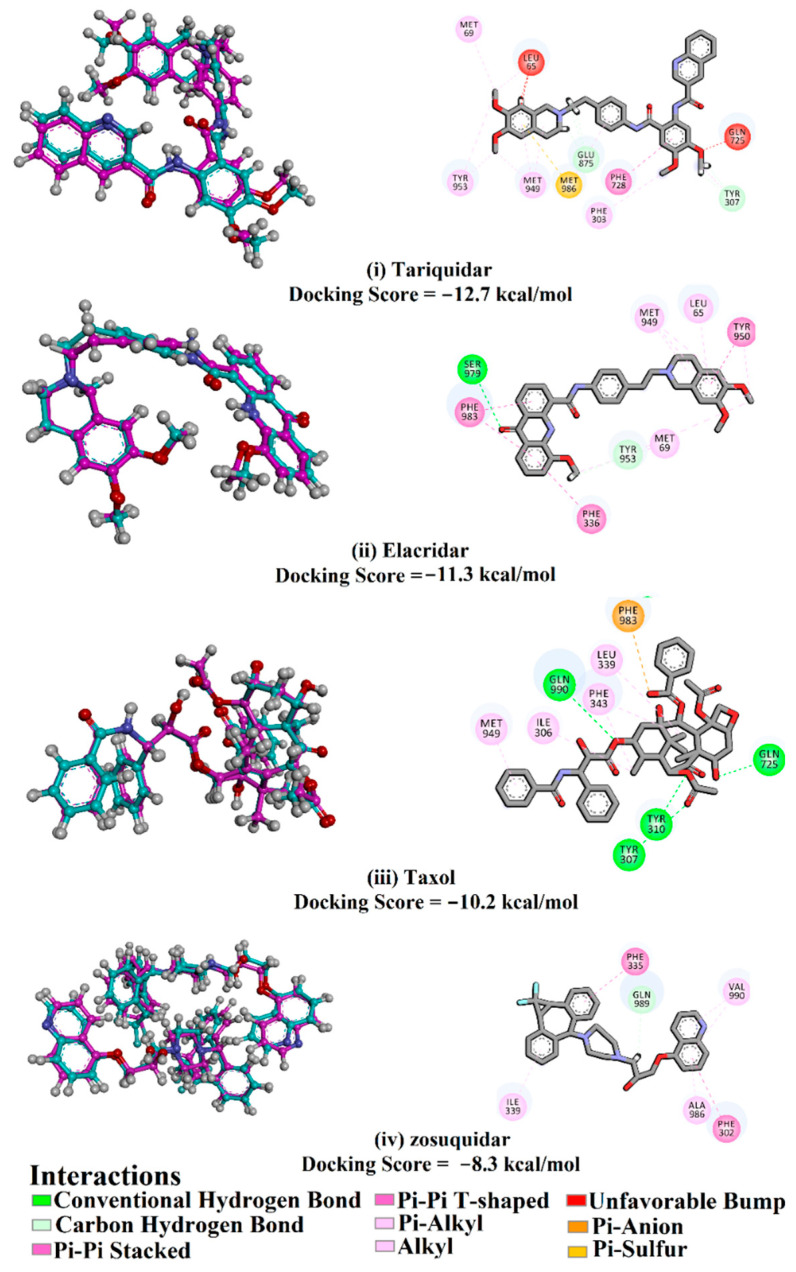
3D and 2D molecular interactions of the experimental structures (in cyan) and the portended docking poses (in pink) of (**i**) tariquidar, (**ii**) elacridar, (**iii**) taxol, and (**iv**) zosuquidar in complex with ABCB1 transporter. The predicted docking scores are displayed in kcal/mol.

The AutoDock4.2.6 protocol was initially appraised according to obtainable experimental data. The co-crystallized taxol, zosuquidar, tariquidar, and elacridar inhibitors complexed with ABCB1 transporter (PDB codes: 6qex [17], 6qee [17], 7a6e [18], and 7a6c [18], respectively) were investigated and the portended docking poses were compared with the native co-crystallized complexes (Figure 1).

The comparison between anticipated docked structures and the native co-crystallized complexes exposed that the AutoDock4.2.6 software minutely foretold the splendid docking poses of taxol, zosuquidar, tariquidar, and elacridar inside the binding site of the ABCB1 transporter (Figure 1). The computed root-mean-square deviation (RMSD) between the portended docked and native co-crystallized complexes were 0.23, 0.36, 0.45, and 0.18 Å for taxol, tariquidar, elacridar, and zosuquidar, respectively. Based on the expected docking scores, tariquidar demonstrated the highest binding affinity with a docking score of −12.7 kcal/mol, pursued by elacridar, taxol, and zosuquidar with docking scores of −11.3, −10.2, and −8.3 kcal/mol, respectively. Taxol exhibits four essential hydrogen bonds with GLN725, TYR307, GLN347, and GLN990 with bond lengths of 1.93, 2.27, 1.93, and 2.91 Å, respectively. Furthermore, elacridar exhibits a fundamental hydrogen bond with SER979, with a bond length of 3.12 Å. Notwithstanding, tariquidar and zosuquidar were not capable of exhibiting any hydrogen bond within the binding site; however, their surpassing binding affinities may be ascribed to other types of interaction like van der Waals and hydrophobic interactions (Figure 1).

Further investigations involving molecular dynamics (MD) simulations and binding affinity computations were carried out to probe the stability and consistency of co-crystallized ligands within the binding pocket of the ABCB1 transporter.

### 2.2. Molecular Dynamics of Co-Crystallized Ligands

**Figure 2:**
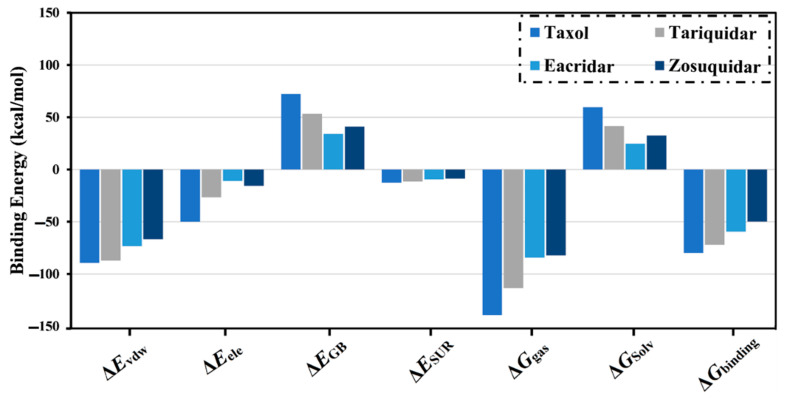
Components of the MM-GBSA approach for taxol, tariquidar, elacridar, and zosuquidar complexed with ABCB1 transporter over the 100 ns MD simulations.

In an attempt to obtain further reliability in the in silico results, MD simulations were executed throughout the 100 ns for taxol, tariquidar, elacridar, and zosuquidar in complex with ABCB1 transporter. Besides, the corresponding binding affinities (∆*G*_binding_) were calculated throughout the 100 ns MD simulations (Figure 2). As shown in Figure 2, the average MM-GBSA binding energies of taxol, tariquidar, elacridar, and zosuquidar were −79.7, −72.0, −59.7, and −49.8 kcal/mol, respectively. The current results warrant the great binding affinity of taxol over tariquidar, elacridar, and zosuquidar as an ABCB1 transporter inhibitor.

To investigate the nature of co-crystallized ligand interactions with the ABCB1 transporter, decomposition of binding energies was carried out (Figure 2). Moreover, analysis of binding energy components for taxol, tariquidar, elacridar, and zosuquidar manifested the most influential contribution of van der Waals *(E*_vdw_) interactions with an average value of −89.4, −87.1, −73.4, and −66.7 kcal/mol, respectively. As well, electrostatic *(E*_ele_) interactions of taxol, tariquidar, elacridar, and zosuquidar in complex with ABCB1 transporter were appropriate (calc. −49.4, −26.5, −11.0, and −15.6 kcal/mol, respectively). Notably, *E*_vdw_ interactions for taxol and tariquidar were approximately the same. Nevertheless, the *E*_ele_ interactions for taxol were more favorable than tariquidar, which is in line with the number of hydrogen bonds formed with the ABCB1 transporter.

As a result, the NPASS database was virtually screened to identify novel inhibitors against the ABCB1 transporter, and taxol was considered as a reference inhibitor in the next calculations.

### 2.3. Virtual Screening of NPASS Database

To explore potent inhibitors separated from a natural resource to struggle multidrug resistance (MDR), the NPASS database was screened against the ABCB1 transporter using the molecular docking technique. Three stages of molecular docking estimations were accomplished to slash computation cost and time. At the outset, all NPASS database was screened towards the ABCB1 transporter with fast docking parameters of *eval*  =  2,500,000 and *GA*  =  25. The NPASS molecules were subsequently ranked on the basis of their docking scores. According to the computed docking scores, approximately a quarter of the filtrated NPASS compounds (≈25%) manifested docking scores lower than −8.0 kcal/mol with the ABCB1 transporter. The top 8668 NPASS compounds from the fast screening were opted and redocked towards the ABCB1 transporter with a moderate docking protocol. Based on the calculated moderate docking scores, the top 866 (≈10%) NPASS compounds were then submitted to expensive molecular docking estimations. The evaluated docking scores for the top 866 NPASS compounds against the ABCB1 transporter are displayed in Table S1. It is worth mentioning that molecular docking calculations with expensive parameters would give more reliable docking scores compared to the moderate and fast docking parameters.

As shown in Table S1, more than three-fourths of the filtrated NPASS compounds (≈83%) demonstrated docking scores less than that of the reference inhibitor (taxol = −10.2 kcal/mol) with ABCB1 transporter. Calculated docking scores, 2D chemical structures, and the binding features of nine potent NPASS compounds with the ABCB1 transporter are summarized in Table 1. Besides, the 3D and 2D representations of interactions of the nine potent NPASS compounds with the proximal amino acids of the ABCB1 transporter are depicted in Figure S1. Notably, those nine NPASS compounds were nominated according to further energetic estimations in the latter sections. What is interesting about the binding features in Table 1 and Figure S1 is that most of the scrutinized NPASS compounds elucidated almost identical docking poses, exhibiting a substantial hydrogen bond with GLN990, except NPC70862. Other interactions were ditto monitored, like van der Waals, pi-based, and hydrophobic interactions between the identified NPASS compounds and the ABCB1 transporter (Figure S1).

**Figure 3:**
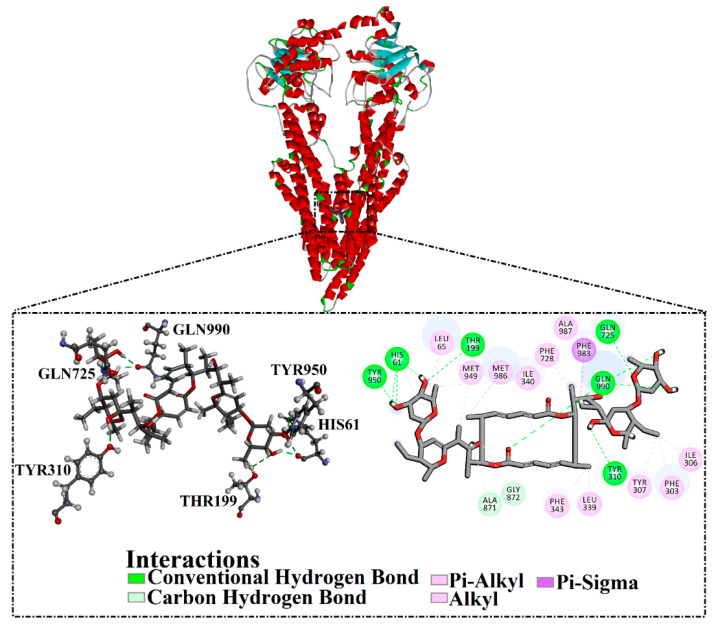
3D and 2D molecular interactions of the predicted binding mode of NPC197736 within the ABCB1 transporter.

Compound NPC197736 unveiled a superb binding affinity towards the ABCB1 transporter with a docking score of −13.5 kcal/mol (Table 1). The outstrip potentiality of NPC197736 as an ABCB1 inhibitor may be ascribed to its ability to display several hydrogen bonds, van der Waals, and hydrophobic interactions with the fundamental amino acids within the binding pocket of the ABCB1 transporter (Figure 3). More minutely, structural insights into the docking pose of the NPC197736 inside the ABCB1 transporter disclosed that the six hydroxyl groups of tetrahydro-2H-pyran-3,4-diol rings exhibit seven hydrogen bonds with the carbonyl GLN990 (1.99, 2.08 Å), the hydroxyl group of TYR950 (2.27 Å), imidazole ring and the carbonyl group of HIS61 (2.71, 1.90, 2.29 Å) and the hydroxyl group of THR190 (2.95 Å) (Figure 3). Besides, a carbonyl group of NPC197736 interacts with the NH2 group of GLN990 with a bond length of 2.36 Å (Figure 3). While the hydroxyl group of tetrahydro-2H-pyran-2-ol forms a hydrogen bond with the hydroxyl group of TYR310 with a bond length of 1.90 Å (Figure 3). Ultimately, the oxygen atom of the tetrahydro-2H-pyran-3,4-diol ring displays a hydrogen bond with NH2 of GLN725 (2.34 Å) (Figure 3).

### 2.4. Molecular Dynamics Simulations

**Figure 4:**
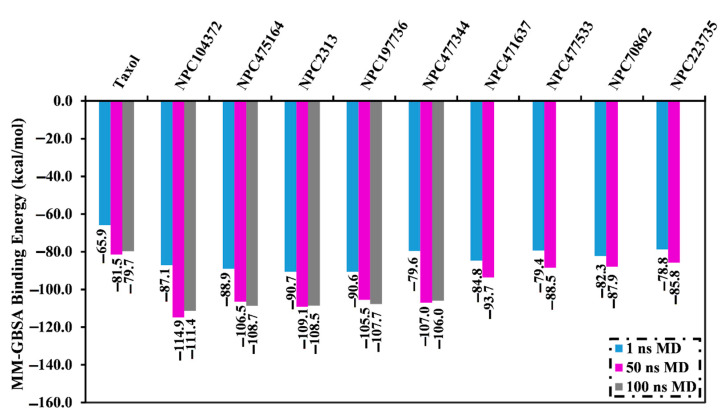
Computed binding affinities for taxol and the top nine potent NPASS compounds complexed with the active site of ABCB1 transporter over 1 ns implicit-solvent MD in addition to 50 ns and 100 ns explicit-solvent MD simulations.

Molecular dynamics (MD) simulations grasp structural minutiae, stabilities of inhibitor-receptor complexes, the thoroughness of receptor-inhibitor binding energies, in addition to configurational flexibilities [19,20]. Therefore, MD simulations and the corresponding binding energy estimations were conducted for the top 10% of the screened NPASS compounds (i.e., 86 molecules) complexed with the ABCB1 transporter. In order to diminish the in silico cost and time, the MD simulations were carried out in the implicit-solvent over 1 ns. MM-GBSA method was employed to compute the binding free energies. The evaluated MM-GBSA binding energies for the opted NPASS compounds are summarized in Table S2. As shown in Table S2, nine NPASS compounds exposed lower binding affinities (Δ*G*_binding_) than that of taxol (calc. −65.9 kcal/mol). Those potent nine NPASS compounds were then submitted to 50 ns MD simulations in an explicit-solvent to attain more trustworthy binding affinities of the NPASS compounds in complex with the ABCB1 transporter. Additionally, the corresponding binding affinities were computed and presented in Figure 4. What is striking about the data in Figure 4 is that about half of NPASS compounds (i.e., 5 molecules) unveiled greater binding affinities (Δ*G*_binding_) than that of taxol (calc. −81.5 kcal/mol). As a consequence, those potent NPASS compounds were chosen and adopted to 100 ns MD simulations in the explicit-solvent to enhance the accuracy of the noticed findings. Besides, the corresponding binding energies were computed (Figure 4). It is apparent that there was no discrepancy between the evaluated MM-GBSA binding affinity for NPC104372, NPC475164, NPC2313, NPC197736, and NPC477344 complexed with ABCB1 transporter throughout the 50 ns and 100 ns MD simulations (Figure 4). Compared to taxol (Δ*G*_binding_ = −79.7 kcal/mol), NPC104372, NPC475164, NPC2313, NPC197736, and NPC477344 demonstrated better binding affinity throughout the 100 ns MD simulations against ABCB1 transporter with an average Δ*G*_binding_ of −111.4, −108.7, −108.5, −107.7, and −106.0 kcal/mol, respectively (Figure 4). 

The estimated MM-GBSA binding energies were then decomposed into separate items towards a better comprehension of the dominant interactions between NPASS compounds and the ABCB1 transporter. For compounds NPC104372, NPC475164, NPC2313, NPC197736, NPC477344, and taxol, binding affinities were governed by *E*_vdw_ interactions with a value ranging between −89.4 and −110.1 kcal/mol. Besides, *E*_ele_ interactions were adequate, with values ranging from −49.9 to −83.6 kcal/mol (Table 2).

**Figure 5:**
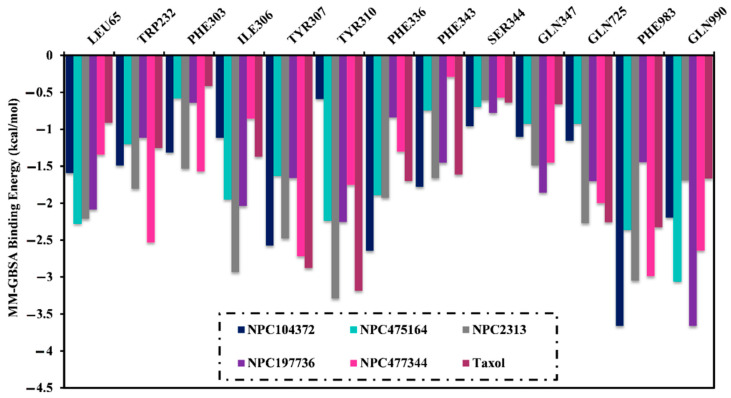
Per-residue energy decomposition of NPC104372, NPC475164, NPC2313, NPC197736, NPC477344, and taxol complexed with ABCB1 transporter.

Furthermore, to probe the dominant amino acid residues that demonstrate preeminent participations to NPC104372-, NPC475164-, NPC2313-, NPC197736-, NPC477344-, and taxol-ABCB1 interactions, the per-residue energy decomposition was performed. All the amino acid residues with energetical participation less than −0.50 kcal/mol were taken into consideration and depicted in Figure 5. It can be seen from the data in Figure 5 that TYR307, TYR310, GLN725, and GLN990 amino acids shared the interactions of NPC104372, NPC475164, NPC2313, NPC197736, NPC477344, and taxol with the ABCB1 transporter. Appreciable participation of the GLN990 amino acid to the total binding affinity was noticed with a value ranging between −1.7 and −3.7 kcal/mol (Figure 5).

### 2.5. Post-Dynamics Analyses 

Structural and energetical analyses were accomplished through the MD course of 100 ns in order to affirm the constancy and demeanor of NPC104372, NPC475164, NPC2313, NPC197736, and NPC477344 complexed with ABCB1 transporter. 

#### 2.5.1. Hydrogen Bond Analysis

**Figure 6:**
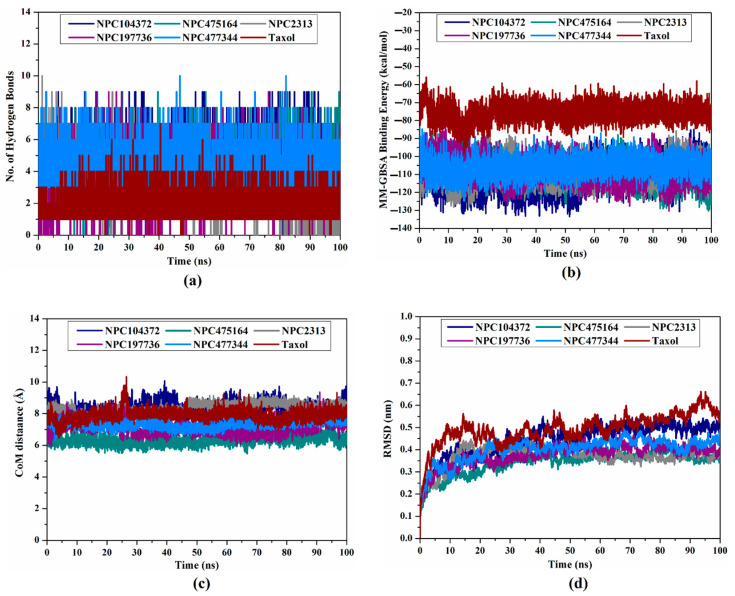
(**a**) The number of hydrogen bonds between the identified NPASS compounds and ABCB1 transporter, (**b**) evaluated MM-GBSA binding energy per frame, (**c**) center-of-mass (CoM) distances, and (**d**) root-mean-square deviation (RMSD) of the backbone atoms from the starting structure of NPC104372 (in navy), NPC475164 (in cyan), NPC2313 (in gray), NPC197736 (in light blue), and taxol (in wine) towards ABCB1 transporter throughout 100 ns MD simulations.

Hydrogen bond analysis was executed to estimate the constancy of hydrogen bonds between the discovered NPASS compounds and the ABCB1 transporter throughout the 100 ns MD simulations. The number of hydrogen bonds in each collected trajectory was computed and illustrated in Figure 6a. As shown in Figure 6a, the average number of hydrogen bonds between the NPASS compounds and the ABCB1 transporter ranged between 3 and 5. The number of hydrogen bonds in NPASS-ABCB1 complexes was greater than that of taxol (calc. 2), resulting in the superior binding affinity of these NPASS compounds compared to taxol.

#### 2.5.2. Binding Energy Per Frame

The steadiness of the identified potent NPASS compounds within the binding site of the ABCB1 transporter was examined by means of investigating the correlation between the binding energy per frame and time (Figure 6b). The most interesting aspect of this graph is the comprehensive stabilization of the five discovered inhibitors and taxol during 100 ns MD simulations with average binding energies (Δ*G*_binding_) ranging from −79.7 to −111.4 kcal/mol (Figure 6b). The most surprising aspect of the analysis is that all deliberated systems preserve constancy throughout 100 ns MD simulations.

#### 2.5.3. Center-of-Mass Distance 

To allow a deeper insight into the steadiness of NPASS-ABCB1 over the MD simulations, center-of-mass (CoM) distances were measured between NPC104372, NPC475164, NPC2313, NPC197736, NPC477344, and taxol and GLN990 residue (Figure 6c). Looking at Figure 6c, it is apparent that CoM distances were more narrow-fluctuated for NPC104372, NPC475164, NPC2313, NPC197736, and NPC477344 complexed with ABCB1 transporter than taxol-ABCB1 complex, with average values of 8.3, 6.3, 8.3, 7.1, 7.3, and 7.9 Å, respectively. The most significant finding was that NPC104372, NPC475164, NPC2313, NPC197736, and NPC477344 bound tightly with the ABCB1 transporter.

#### 2.5.4. Root-Mean-Square Deviation

To explore the conformation dynamics of the NPC104372-, NPC475164-, NPC2313-, NPC197736-, NPC477344-, and taxol-ABCB1 complexes, the root-mean-square deviation (RMSD) values of the backbone atoms of the entire system were evaluated (Figure 6d). Categorically, the evaluated RMSD values for the scrutinized systems stayed beneath 0.6 nm throughout 100 ns MD simulations (Figure 6d). Besides, all investigated complexes had higher fluctuations in the first 10 ns and demonstrated overall stability starting from 20 ns till the end of the 100 ns MD simulations. These findings imply that NPC104372-, NPC475164-, NPC2313-, NPC197736-, NPC477344-, and taxol are tightly bound and have no effect on the ABCB1 transporter’s topology.

### 2.6. Water Solubility of the Identified NPs

One of the most important properties influencing drug absorption is solubility [21]. Therefore, the water solubility of the identified NPS was predicted with the assistance of the PkCSM online tool (http://biosig.unimelb.edu.au/pkcsm/prediction (accessed on 1 May 2022). The estimated molar solubility in water (log S) was −2.8, −2.9, −3.0, −3.0, −2.8, and −2.9 mol/L for NPC104372-, NPC475164-, NPC2313-, NPC197736-, NPC477344, and taxol, respectively. It is worth noting that the identified hits demonstrate strong solubility in water.

## 3. Computational Methodology

### 3.1. ABCB1 Preparation

The cryo-electron microscopy (EM) resolved structure of the human ABCB1 (PDB code: 6qex [17]) was picked out as a template for all in silico computations. Extracellular domains, crystalline water molecules, ions, and ligand were removed. Therefore, H++ webserver was employed to deliberate the protonation states of the titratable amino acid residues [22]. Besides, all missing hydrogen atoms were added.

### 3.2. Assessment of Molecular Docking Protocol

In a recent study, the performance of the employed molecular docking protocol was assessed based on four cryo-electron microscopy (EM) resolved ABCB1 transporter in complex with inhibitor. The four inhibitors were [(1*S*,2*S*,3*R*,4*S*,7*R*,9*S*,10*S*,12*R*,15*S*)-4,12-diacetyloxy-15-[(2*R*,3*S*)-3-benzamido-2-hydroxy-3-phenylpropanoyl]oxy-1,9-dihydroxy-10,14,17,17-tetramethyl-11-oxo-6-oxatetracyclo[1 1.3.1.03,10.04,7]heptadec-13-en-2-yl] benzoate (taxol), (2*R*)-1-[4-[(2*S*,4*R*)-3,3-difluoro-11-tetracyclo[10.4.0.0^2,4^.0^5,10^]hexadeca-1(16),5,7,9,12,14-hexaenyl]piperazin-1-yl]-3-quinolin-5-yloxypropan-2-ol (zosuquidar), *N*-[2-[[4-[2-(6,7-dimethoxy-3,4-dihydro-1H-isoquinolin-2-yl)ethyl]phenyl]carbamoyl]-4,5-dimethoxyphenyl]quinoline-3-carboxamide (tariquidar), and *N*-[4-[2-(6,7-dimethoxy-3,4-dihydro-1*H*-isoquinolin-2-yl)ethyl]phenyl]-5-methoxy-9-oxo-10*H*-acridine-4-carboxamide (elacridar). The 3D structures of taxol, zosuquidar, tariquidar, and elacridar were obtained from resolved structures (PDB codes: 6qex [17], 6qee [17], 7a6e [18], and 7a6c [18], respectively). 

### 3.3. Database Preparation

The Natural Product Activity and Species Source (NPASS) database, including 35,032 molecules, was downloaded and prepared [23]. All molecules were gained in 2D structural data format (SDF). Omega2 software was employed to create the 3D chemical structures of all NPASS compounds and various conformations within an energy window of 10 kcal/mol were created for each compound [24,25]. MMFF94S force field within SZYBKI software was utilized to optimize the geometrical structures of the NPASS molecules [26,27]. The partial atomic charges of NPASS molecules were computed with the assistance Gasteiger-Marsili method [28]. Duplicated molecules with congruent InChIKey were eliminated [29]. The number of duplicates was 1111 molecules. The prepared files of the NPASS database may be available by means of www.compchem.net/ccdb (accessed on 1 May 2022).

### 3.4. Molecular Docking

All molecular docking computations were fulfilled utilizing the AutoDock4.2.6 software [30]. The ABCB1 structure was processed using MGtools1.5.6. Besides, the pdbqt file was created according to the AutoDock4.2.6 protocol [31]. Fast, moderate, and expensive docking computations were executed, in which the number of the Lamarckian genetic algorithm (*GA*) runs and the maximum number of energy evaluations (*eval*) run variables were adjusted to 25 and 2,500,000, 100 and 10,000,000 and 250 and 25,000,000, respectively. Other docking settings were maintained as default. The docking grid dimensions were designated to encompass the binding pocket of the ABCB1 protein (60 Å × 60 Å × 60 Å) and the spacing value was 0.375 Å. As well, the coordinates of the grid center were specified as 177.419, 167.849, and 153.194 (in *x*, *y*, and *z* dimensions, respectively). The predicted docking poses for each NPASS compound were clustered using the built-in clustering analysis with an RMSD tolerance of 1.0 Å. The docking pose of the lowest energy within the highest cluster was chosen as a representative pose.

### 3.5. Molecular Dynamics Simulations

Molecular dynamics (MD) simulations were performed for the most potent NPASS compounds in complex with the ABCB1 transporter with the assistance of AMBER16 software [32]. The general AMBER force field (GAFF2) was employed to parameterize NPASS compounds [33], while the ABCB1 transporter was parameterized using an AMBER force field of 14SB [34]. The minutiae of employed MD simulations are characterized in [35,36,37]. In this work, both implicit- and explicit-solvent MD simulations were executed.

In the implicit-solvent MD simulations, the atomic partial charges of NPASS compounds were assigned utilizing an AM1-BCC method [38]. No cutoff and periodic boundary conditions were employed for nonbonded interactions. The aqueous solvation effect was deemed via employing igb = 1 solvent model [39]. For all the docked NPASS compounds complexed with the ABCB1 transporter, energy minimization was firstly performed for 500 steps. After that, the minimized complexes were smoothly heated up to 300 K utilizing a constant volume periodic boundary (NVT) for 10 ps. Systems were subsequently equilibrated for 50 ps. The production run was afterward executed for 1 ns MD simulations. In explicit-solvent MD simulations, the restrained electrostatic potential (RESP) fitting approach was applied to determine the charges of the scrutinized NPASS compounds at the HF/6-31G* level with the aid of Gaussian09 software [40,41]. A water-solvated octagonal box was built utilizing a TIP3P water model [42]. The 0.15 M ions (Na^+^ and Cl^−^) were inserted to supply charge neutralization. The solvated systems were initially minimized utilizing 5000 steps of steepest descent and then 5000 steps of conjugate gradient algorithm. Afterward, the minimized systems were gently heated from 0 to 300 k throughout 50 ps. Besides, the complexes were appropriately equilibrated for 1 ns. The equilibrated systems underwent an extra productive MD run over simulation times of 50 ns and 100 ns under an NPT ensemble for each investigated NPASS-ABCB1 complexes. Snapshots were recorded every 10 ps for the post-dynamics analyses and binding energy calculations. The Particle Mesh Ewald (PME) algorithm was employed to handle the long-range electrostatic interactions [43]. A Langevin thermostat equilibration scheme (ntt  = 3) was employed to preserve the temperature at 298 k utilizing a collision frequency gamma_ln of 1.0 [44]. The Berendsen barostat was utilized for the pressure control with 2 ps relaxation time [45]. SHAKE algorithm with a time step of 2 fs was applied to restrain all hydrogen-involved bonds [46]. All MD simulations were conducted with the assistance of the GPU version of pmemd (pmemd.cuda) within the AMBER16 package.

### 3.6. MM-GBSA Binding Energy Estimations

The binding energy estimations of the selected NPASS compounds complexed with the ABCB1 transporter were evaluated utilizing the molecular mechanical-generalized Born surface area (MM-GBSA) method [47]. In this work, the polar desolvation free energy (Δ*G*_GB_) was assigned utilizing the modified generalized Born (GB) model suggested via Onufriev et al. (igb = 2) [48]. According to the gathered snapshots during the MD course, the absolute binding energy (Δ*G*_binding_) was estimated according to the following equation:

$$\Delta G_{\mathrm{binding}}=G_{Complex\;}-\left(G_{\mathrm{NPASS}}+G_{\mathrm{ABCB}1}\right)$$

where

$$G=E_{\mathrm{vdw}}+E_{\mathrm{ele}}+G_{\mathrm{GB}}+G_{\mathrm{SA}}$$

The terms *G*_SA_, *E*_ele_, *G*_GB_, and *E*_vdw_ represent the surface area, electrostatic, general Born solvation, and van der Waals energies, respectively. The configurational entropy (S) was neglected. All molecular docking calculations, MD simulations, and quantum mechanics (QM) calculations were performed on the CompChem GPU/CPU hybrid cluster (hpc.compchem.net (accessed on 18 March 2022)). BIOVIA DS Visualize 2020 was applied to visualize the 3D and 2D representations of the ABCB1-NPASS interactions [49].

## 4. Conclusions

ABCB1 is one of the most critical targets for conquering the MDR phenomena. In the current study, in silico drug screening techniques were executed for the sake of identifying potential NPs from the NPASS database that will be able to inhibit the ABCB1 transporter. MD simulations were conducted based on the expected docking scores and the NPASS-ABCB1 binding energies were calculated. MM-GBSA binding energies throughout MD simulations over 100 ns demonstrated that NPC104372, NPC475164, NPC2313, NPC197736, and NPC477344 displayed auspicious binding affinities against the ABCB1 transporter with Δ*G*_binding_ of −111.4, −10.8.7, −108.5, −107.7, and 106.0 kcal/mol, respectively. The stabilities of the identified NPASS compounds were further demonstrated using the structural and energetical analyses of the investigated complexes over the 100 ns MD course. In summary, these recent findings confirm that NPC104372, NPC475164, NPC2313, NPC197736, and NPC477344 are effective inhibitors of the ABCB1 transporter and promising for in vitro and in vivo assays.

## Figures and Tables

**Table 1:** Anticipated fast, moderate, and expensive docking scores (in kcal/mol), 2D chemical structures, as well as binding features for the top nine up-and-coming NPASS compounds against ABCB1 transporter. ^a^

No.	NPASS Code	2D Chemical Structure	Docking Score (kcal/mol)			Binding Features ^b^
			Fast	Moderate	Expensive	
	Taxol	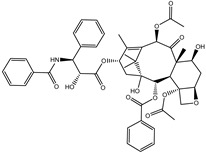	−9.4	−8.0	−10.2	TYR307 (2.27 Å), GLN347 (3.29 Å), GLN725 (1.93 Å), GLN990 (2.91 Å)
1	NPC197736	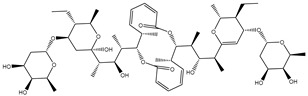	−8.8	−10.9	−13.5	HIS61 (1.90, 2.29, 2.71 Å), THR199 (2.95 Å), TYR310 (1.90 Å), GLN725 (2.34 Å), TYR950 (2.27 Å), GLN990 (1.99, 2.08, 2.36 Å)
2	NPC477533	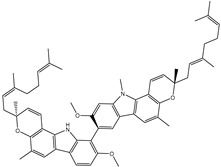	−11.1	−10.9	−13.3	GLN990 (2.24 Å)
3	NPC223735	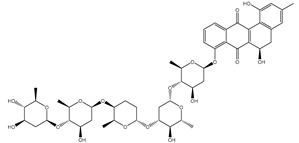	−9.9	−11.4	−12.6	TYR310 (2.64 Å), GLN347 (2.01, 3.08 Å), GLN725 (2.03 Å), GLN946 (2.05 Å), GLN990 (2.22 Å)
4	NPC70862	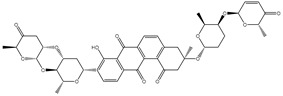	−11.6	−11.8	−12.2	ASN842 (2.57, 2.79 Å), TYR950 (1.74 Å)
5	NPC471637	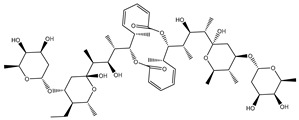	−9.7	−11.4	−12.2	THR199 (1.62 Å), TRP232 (2.36 Å), TYR307 (2.03 Å), GLN725 (2.52, 2.59, 2.85 Å), GLU875 (1.86 Å), GLN990 (2.31 Å)
6	NPC104372	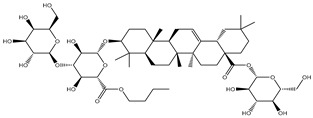	−9.9	−11.0	−12.2	HIS61 (2.15 Å), GLY62 (2.26 Å), GLY64 (2.95 Å), LEU65 (2.30 Å), GLN195 (2.12 Å), THR199 (1.63 Å), SER344 (2.11 Å), GLN946 (3.22 Å)
7	NPC2313	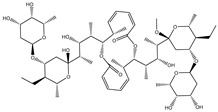	−9.9	−12.1	−12.0	GLY62 (2.93 Å), LEU65 (2.98 Å), GLN195 (2.20, 2.73 Å), TYR310 (2.96 Å), GLN347 (2.35 Å), GLN946 (2.13 Å), GLN990 (1.76, 2.30 Å)
8	NPC475164	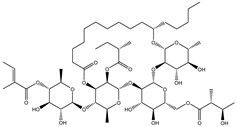	−9.3	−11.3	−11.9	TYR307 (2.08 Å), TYR310 (2.17 Å), GLN347 (3.20 Å), ASN842 (2.16 Å), GLN946 (1.70 Å), MET986 (1.82 Å), GLN725 (3.00 Å), GLN990 (2.61 Å)
9	NPC477344	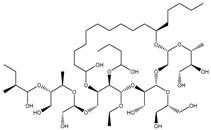	−10.2	−11.1	−11.9	TYR310 (2.86 Å), SER344 (2.21 Å), GLY872 (2.72 Å), GLU875 (1.85 Å), MET986 (1.93 Å), GLN990 (1.89, 2.08, 2.54 Å)

^a^ Data sorted according to the computed expensive molecular docking. ^b^ Conventional hydrogen bond only (in Å) is listed.

**Table 2:** Estimated MM-GBSA binding energies of NPC104372, NPC475164, NPC2313, NPC197736, NPC477344, and taxol within the ABCB1 binding pocket throughout the 100 ns MD simulations.

NPASS Code	Estimated MM-GBSA Binding Energy (kcal/mol)						
	∆*E*_vdw_	∆*E*_ele_	∆*E*_GB_	∆*E*_SUR_	∆*G*_gas_	∆*G*_Solv_	∆*G*_binding_
NPC104372	−110.1	−83.6	98.1	−15.8	−193.6	82.2	−111.4
NPC475164	−120.9	−64.3	93.9	−17.4	−185.2	76.5	−108.7
NPC2313	−119.0	−65.6	92.3	−16.2	−184.6	76.1	−108.5
NPC197736	−120.8	−60.2	89.8	−16.5	−181.0	73.3	−107.7
NPC477344	−111.4	−76.7	98.6	−16.4	−188.2	82.2	−106.0
Taxol	−89.4	−49.9	72.2	−12.6	−139.3	59.6	−79.7

## Data Availability

The data presented in this study are available in the Supplementary Material.

## Supplementary Materials

The following supporting information can be downloaded at: https://www.mdpi.com/article/10.3390/molecules27103104/s1, Figure S1: 3D and 2D representations of the binding modes of the nine potent molecules and taxol complexed with ABCB1 transporter; Table S1: Estimated fast, moderate, and expensive docking scores for taxol and the top 866 potent NPASS compounds within the ABCB1 binding pocket; Table S2: Estimated docking scores and MM-GBSA binding energies (in kcal/mol) over 1 ns implicit-solvent MD simulations for taxol and the top 86 NPs within the P-gp binding pocket.
