# Supplementary material for: Exploring Natural Product Activity and Species Source Candidates for Hunting ABCB1 Transporter Inhibitors: An In Silico Drug Discovery Study
Source: Molecules. 2022 May 12;27(10):3104. doi: 10.3390/molecules27103104 (PMC9143904; doi:10.3390/molecules27103104)
Supplement: Supplementary file 1 [file molecules-27-03104-s001.zip › molecules-1666383-supplementary.pdf]

# Exploring Natural Product Activity and Species Source Candidates for Hunting ABCB1 Transporter Inhibitors: An *In Silico* Drug Discovery Study

**Mahmoud A. A. Ibrahim [1,*], Khlood A. A. Abdeljawaad [1], Alaa H. M. Abdelrahman [1], Laila A. Jaragh-Alhadad [2], Hesham Farouk Oraby [3,4], Eslam B. Elkaeed [5], Gamal A. H. Mekhemer [1], Gamal A. Gabr [6,7], Ahmed M. Shawky [8], Peter A. Sidhom [9], Mahmoud E. S. Soliman [10], Mahmoud F. Moustafa [11,12], Paul W. Paré [13] and Mohamed-Elamir F. Hegazy [14,*]**

1 Computational Chemistry Laboratory, Chemistry Department, Faculty of Science, Minia University, Minia 61519, Egypt; kh.abdeljawaad@compchem.net (K.A.A.A.); a.abdelrahman@compchem.net (A.H.M.A.); gmekhemer@mu.edu.eg (G.A.H.M.)
2 Department of Chemistry, Faculty of Science, Kuwait University, Safat 13060, Kuwait; laila.alhadad@ku.edu.kw
3 Deanship of Scientific Research, Umm Al-Qura University, Makkah 21955, Saudi Arabia; hforaby@uqu.edu.sa
4 Department of Crop Science, Faculty of Agriculture, Zagazig University, Zagazig 44519, Egypt
5 Department of Pharmaceutical Sciences, College of Pharmacy, AlMaarefa University, Riyadh 13713, Saudi Arabia; ikaeed@mcst.edu.sa
6 Department of Pharmacology and Toxicology, College of Pharmacy, Prince Sattam Bin Abdulaziz University, Al-Kharj 11942, Saudi Arabia; g.gabr@psau.edu.sa
7 Agricultural Genetic Engineering Research Institute (AGERI), Agricultural Research Center, Giza 12619, Egypt
8 Science and Technology Unit (STU), Umm Al-Qura University, Makkah 21955, Saudi Arabia; amesmail@uqu.edu.sa
9 Department of Pharmaceutical Chemistry, Faculty of Pharmacy, Tanta University, Tanta 31527, Egypt; peter.ayoub@pharm.tanta.edu.eg
10 Molecular Bio-Computation and Drug Design Research Group, School of Health Sciences, University of KwaZulu-Natal, Westville, Durban 4000, South Africa; soliman@ukzn.ac.za
11 Department of Biology, College of Science, King Khalid University, Abha 9004, Saudi Arabia; mfmostfa@kku.edu.sa
12 Department of Botany and Microbiology, Faculty of Science, South Valley University, Qena 83523, Egypt
13 Department of Chemistry & Biochemistry, Texas Tech University, Lubbock, TX 79409, USA; paul.pare@ttu.edu
14 Chemistry of Medicinal Plants Department, National Research Centre, 33 El-Bohouth St., Dokki, Giza 12622, Egypt
* Correspondence: m.ibrahim@compchem.net (M.A.A.I.); mohegazy@uni-mainz.de (M.-E. F. H.)

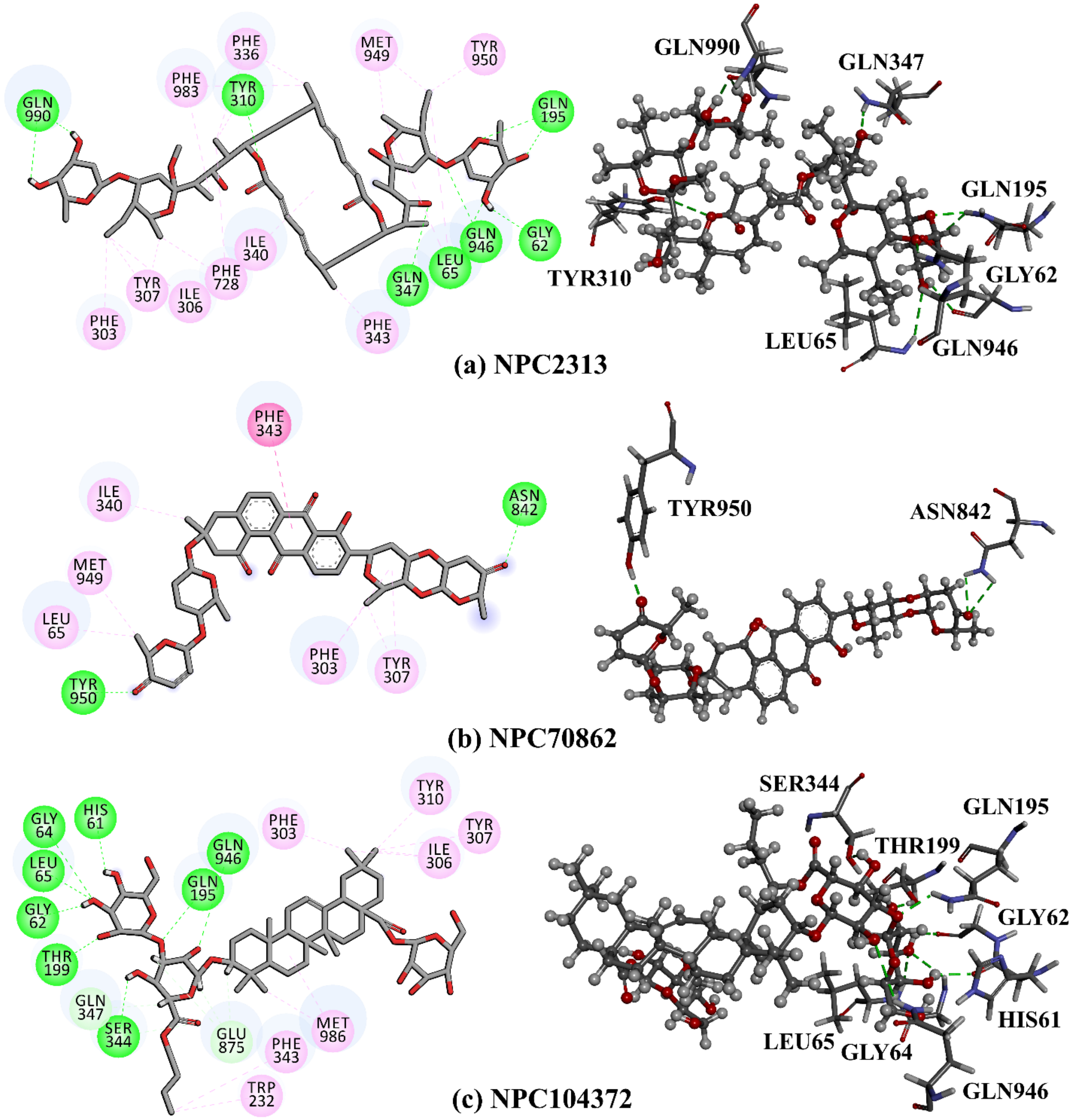


**Figure S1**. 3D and 2D representations of the binding modes of the nine potent molecules and taxol complexed with ABCB1 transporter.

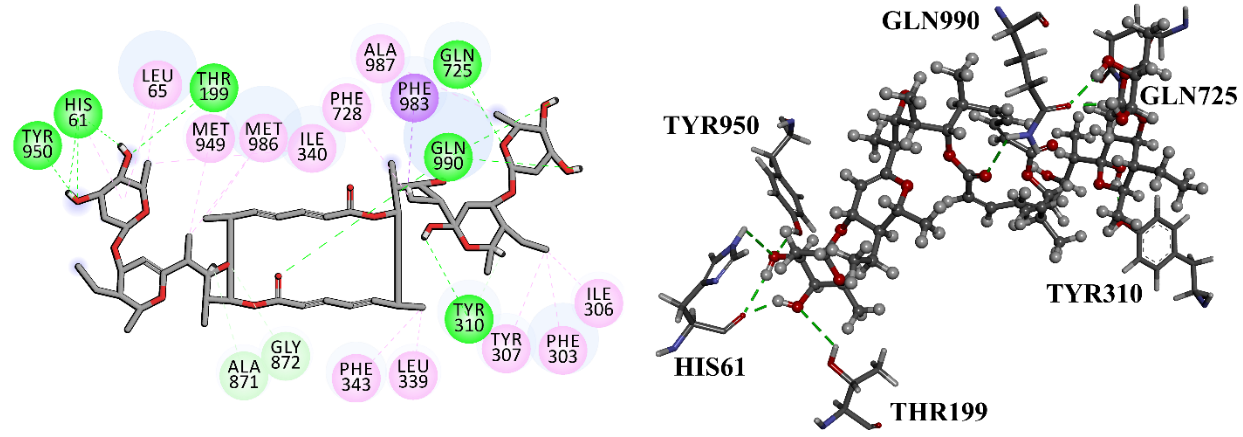


(d) NPC197736

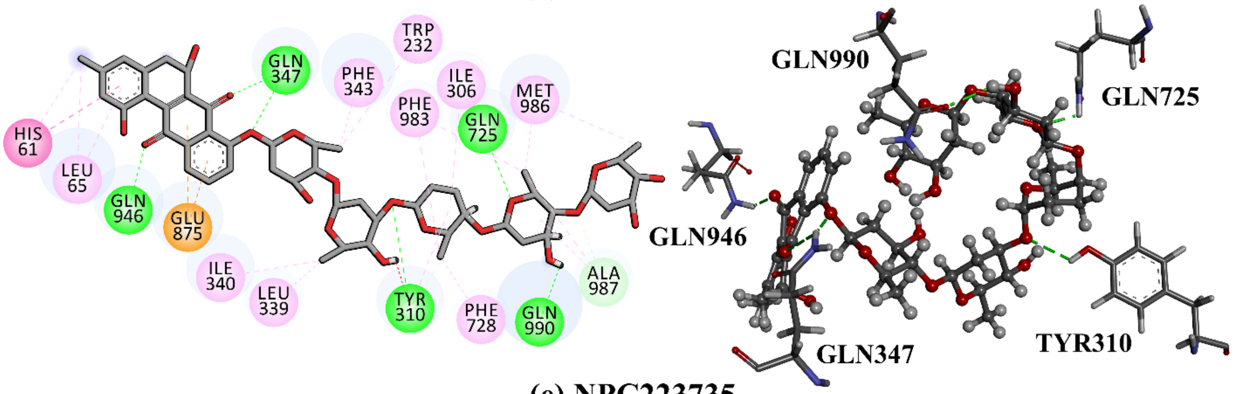


(e) NPC223735

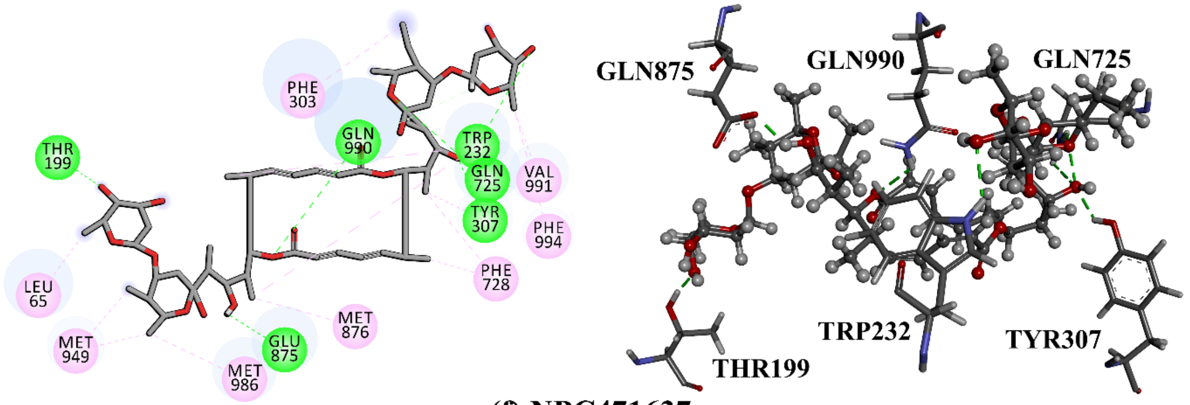


(f) NPC471637

**Figure S1.** *Continued*.

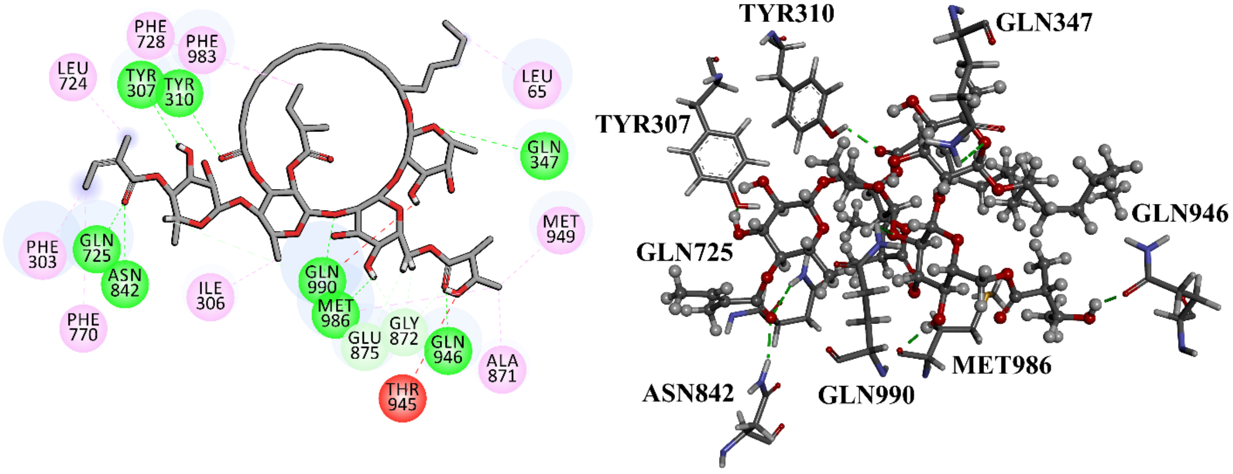


(g) NPC475164

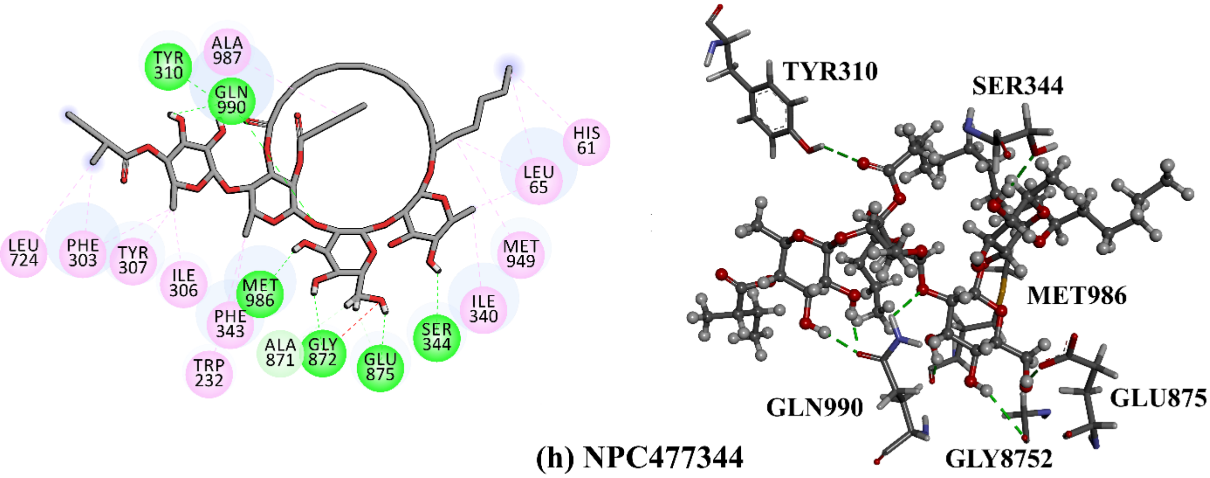


(h) NPC477344

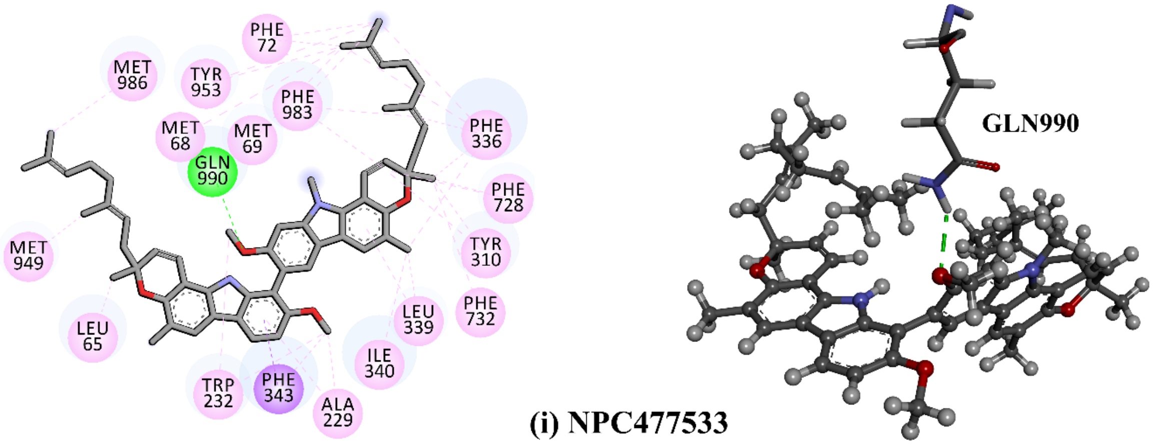


(i) NPC477533

**Figure S1.** *Continued*.

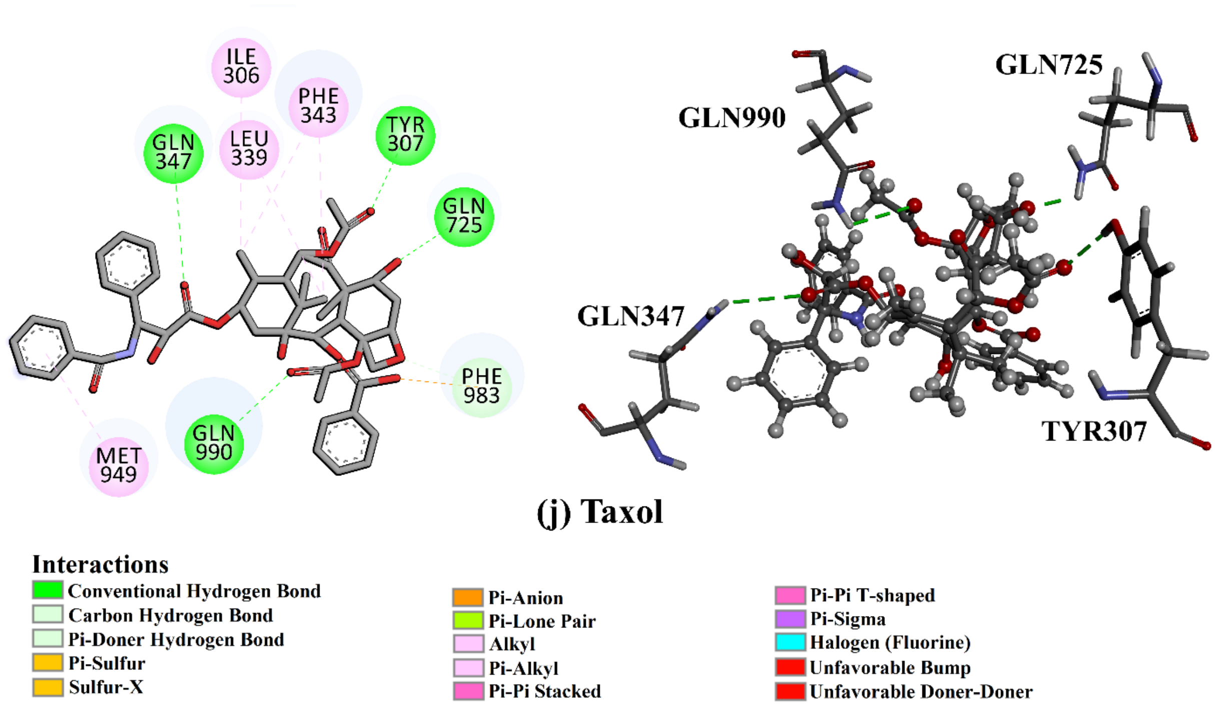


**(j) Taxol**

**Figure S1.** *Continued***.**

**Table S1.** Estimated fast, moderate, and expensive docking scores for taxol and the top 866 potent NPASS compounds within the ABCB1 binding pocket.[a]

| No. | Compound Name/Code | Docking Score (kcal/mol) | | | No. | Compound Name/Code | Docking Score (kcal/mol) | | |
|---|---|---|---|---|---|---|---|---|---|
| | | Fast | Moderate | Expensive | | | Fast | Moderate | Expensive |
| | Taxol | –9.4 | –8.0 | –10.2 | 56 | NPC472937 | –10.3 | –11.4 | –12.1 |
| 1 | NPC276231 | –15.1 | –15.9 | –16.4 | 57 | NPC469671 | –10.3 | –11.8 | –12.1 |
| 2 | NPC470601 | –9.6 | –13.0 | –14.9 | 58 | NPC475655 | –9.6 | –11.1 | –12.1 |
| 3 | NPC470602 | –10.9 | –13.0 | –14.7 | 59 | NPC473612 | –11.7 | –12.0 | –12.0 |
| 4 | NPC472437 | –13.5 | –12.9 | –14.5 | 60 | NPC329943 | –11.4 | –11.8 | –12.0 |
| 5 | NPC242269 | –13.2 | –14.1 | –14.2 | 61 | NPC36754 | –12.0 | –12.0 | –12.0 |
| 6 | NPC475493 | –13.4 | –13.7 | –13.8 | 62 | NPC127197 | –12.0 | –12.0 | –12.0 |
| 7 | NPC197736 | –8.8 | –10.9 | –13.5 | 63 | NPC270958 | –10.2 | –11.4 | –12.0 |
| 8 | NPC477533 | –11.1 | –12.6 | –13.3 | 64 | NPC81137 | –8.8 | –11.9 | –12.0 |
| 9 | NPC202104 | –12.6 | –13.5 | –13.2 | 65 | NPC472936 | –10.6 | –11.5 | –12.0 |
| 10 | NPC208258 | –8.4 | –12.1 | –13.1 | 66 | NPC143173 | –12.0 | –12.0 | –12.0 |
| 11 | NPC231271 | –12.4 | –12.6 | –12.9 | 67 | NPC2313 | –9.7 | –12.1 | –12.0 |
| 12 | NPC472395 | –11.9 | –12.1 | –12.9 | 68 | NPC477874 | –11.7 | –11.8 | –12.0 |
| 13 | NPC473507 | –10.1 | –11.5 | –12.9 | 69 | NPC471858 | –11.5 | –11.5 | –11.9 |
| 14 | NPC470426 | –12.8 | –12.8 | –12.8 | 70 | NPC471863 | –12.1 | –11.9 | –11.9 |
| 15 | NPC224528 | –12.3 | –12.6 | –12.8 | 71 | NPC96605 | –8.5 | –10.6 | –11.9 |
| 16 | NPC6702 | –12.0 | –12.5 | –12.7 | 72 | NPC473547 | –11.9 | –11.9 | –11.9 |
| 17 | NPC475258 | –12.7 | –12.7 | –12.7 | 73 | NPC472934 | –10.7 | –11.3 | –11.9 |
| 18 | NPC65118 | –12.2 | –12.7 | –12.6 | 74 | NPC473720 | –11.8 | –11.9 | –11.9 |
| 19 | NPC477207 | –11.9 | –12.5 | –12.6 | 75 | NPC474581 | –10.6 | –11.6 | –11.9 |
| 20 | NPC223735 | –9.9 | –11.4 | –12.6 | 76 | NPC477344 | –10.2 | –11.1 | –11.9 |
| 21 | NPC471850 | –10.7 | –12.0 | –12.6 | 77 | NPC78159 | –11.3 | –11.0 | –11.9 |
| 22 | NPC126676 | –11.8 | –11.6 | –12.5 | 78 | NPC475164 | –9.3 | –11.3 | –11.9 |
| 23 | NPC183736 | –10.9 | –12.3 | –12.5 | 79 | NPC47905 | –10.8 | –12.2 | –11.9 |
| 24 | NPC74086 | –10.5 | –10.8 | –12.5 | 80 | NPC202428 | –11.2 | –11.8 | –11.9 |
| 25 | NPC471860 | –11.9 | –12.1 | –12.5 | 81 | NPC475520 | –11.9 | –11.8 | –11.9 |
| 26 | NPC123859 | –10.1 | –12.1 | –12.5 | 82 | NPC475570 | –11.7 | –11.8 | –11.8 |
| 27 | NPC307205 | –12.4 | –12.4 | –12.4 | 83 | NPC94531 | –11.0 | –11.5 | –11.8 |
| 28 | NPC327962 | –12.2 | –12.7 | –12.4 | 84 | NPC471583 | –11.8 | –11.8 | –11.8 |
| 29 | NPC472236 | –11.2 | –12.4 | –12.4 | 85 | NPC322800 | –11.2 | –11.8 | –11.8 |
| 30 | NPC473403 | –10.5 | –12.3 | –12.4 | 86 | NPC9856 | –11.8 | –11.8 | –11.8 |
| 31 | NPC133209 | –10.8 | –11.5 | –12.3 | 87 | NPC245756 | –11.7 | –11.8 | –11.8 |
| 32 | NPC469447 | –12.1 | –12.4 | –12.3 | 88 | NPC471382 | –11.8 | –11.8 | –11.8 |
| 33 | NPC471859 | –12.1 | –12.3 | –12.3 | 89 | NPC471891 | –11.8 | –11.8 | –11.8 |
| 34 | NPC69383 | –10.9 | –11.5 | –12.3 | 90 | NPC321661 | –11.2 | –11.7 | –11.8 |
| 35 | NPC231712 | –9.3 | –12.1 | –12.3 | 91 | NPC475197 | –10.4 | –10.4 | –11.8 |
| 36 | NPC239990 | –11.9 | –12.3 | –12.2 | 92 | NPC119068 | –9.6 | –11.7 | –11.8 |
| 37 | NPC472939 | –10.4 | –11.9 | –12.2 | 93 | NPC229234 | –10.6 | –11.1 | –11.8 |
| 38 | NPC250835 | –12.2 | –12.2 | –12.2 | 94 | NPC476451 | –11.8 | –11.8 | –11.8 |
| 39 | NPC471637 | –9.7 | –11.4 | –12.2 | 95 | NPC284888 | –11.7 | –11.8 | –11.8 |
| 40 | NPC104372 | –9.9 | –11.0 | –12.2 | 96 | NPC471053 | –9.5 | –11.5 | –11.8 |
| 41 | NPC313179 | –10.9 | –10.4 | –12.2 | 97 | NPC476517 | –11.3 | –11.7 | –11.7 |
| 42 | NPC4638 | –12.2 | –11.5 | –12.2 | 98 | NPC279442 | –9.3 | –11.7 | –11.7 |
| 43 | NPC162440 | –12.2 | –12.2 | –12.2 | 99 | NPC273755 | –9.7 | –10.4 | –11.7 |
| 44 | NPC65034 | –11.9 | –12.1 | –12.2 | 100 | NPC477873 | –11.2 | –11.4 | –11.7 |
| 45 | NPC177362 | –12.0 | –12.1 | –12.2 | 101 | NPC48992 | –11.4 | –11.6 | –11.7 |
| 46 | NPC70862 | –11.6 | –11.8 | –12.2 | 102 | NPC57586 | –8.7 | –10.7 | –11.7 |
| 47 | NPC208011 | –11.0 | –10.9 | –12.1 | 103 | NPC301941 | –11.8 | –11.9 | –11.7 |
| 48 | NPC266545 | –11.0 | –10.9 | –12.1 | 104 | NPC63047 | –11.7 | –11.7 | –11.7 |
| 49 | NPC470582 | –10.3 | –10.5 | –12.1 | 105 | NPC172311 | –10.2 | –11.6 | –11.7 |
| 50 | NPC472371 | –12.4 | –12.6 | –12.1 | 106 | NPC286994 | –11.6 | –11.7 | –11.7 |
| 51 | NPC313421 | –10.5 | –12.1 | –12.1 | 107 | NPC472807 | –10.9 | –11.7 | –11.7 |
| 52 | NPC477531 | –11.0 | –12.1 | –12.1 | 108 | NPC473575 | –9.5 | –11.2 | –11.7 |
| 53 | NPC103250 | –12.0 | –12.1 | –12.1 | 109 | NPC168135 | –10.3 | –10.7 | –11.7 |
| 54 | NPC149962 | –10.8 | –10.8 | –12.1 | 110 | NPC24599 | –11.7 | –11.7 | –11.7 |
| 55 | NPC105942 | –11.5 | –12.0 | –12.1 | 111 | NPC123319 | –11.1 | –11.5 | –11.7 |

**Table S1.** *Continued*.

| No. | Compound Name/Code | Docking Score (kcal/mol) | | | No. | Compound Name/Code | Docking Score (kcal/mol) | | |
|---|---|---|---|---|---|---|---|---|---|
| | | Fast | Moderate | Expensive | | | Fast | Moderate | Expensive |
| 112 | NPC34580 | –10.9 | –11.7 | –11.7 | 171 | NPC475544 | –9.9 | –10.9 | –11.4 |
| 113 | NPC473398 | –11.6 | –11.6 | –11.7 | 172 | NPC191863 | –11.0 | –11.4 | –11.4 |
| 114 | NPC475658 | –11.7 | –11.7 | –11.7 | 173 | NPC304307 | –10.3 | –10.5 | –11.4 |
| 115 | NPC477065 | –11.5 | –11.6 | –11.7 | 174 | NPC474181 | –11.4 | –11.4 | –11.4 |
| 116 | NPC252133 | –10.7 | –11.5 | –11.6 | 175 | NPC168188 | –9.4 | –11.4 | –11.4 |
| 117 | NPC292706 | –8.9 | –11.6 | –11.6 | 176 | NPC191193 | –10.0 | –10.8 | –11.4 |
| 118 | NPC325032 | –10.1 | –11.6 | –11.6 | 177 | NPC471861 | –11.1 | –11.5 | –11.4 |
| 119 | NPC472574 | –9.2 | –11.4 | –11.6 | 178 | NPC473469 | –9.3 | –10.8 | –11.4 |
| 120 | NPC472694 | –9.8 | –10.9 | –11.6 | 179 | NPC478124 | –11.1 | –11.4 | –11.4 |
| 121 | NPC40455 | –11.0 | –11.6 | –11.6 | 180 | NPC223242 | –9.8 | –11.4 | –11.4 |
| 122 | NPC469507 | –11.5 | –11.6 | –11.6 | 181 | NPC251226 | –10.5 | –11.4 | –11.4 |
| 123 | NPC471782 | –11.5 | –11.6 | –11.6 | 182 | NPC472803 | –11.0 | –11.3 | –11.4 |
| 124 | NPC472027 | –11.6 | –11.6 | –11.6 | 183 | NPC474585 | –9.2 | –11.4 | –11.4 |
| 125 | NPC24748 | –11.6 | –11.6 | –11.6 | 184 | NPC8369 | –11.2 | –11.4 | –11.4 |
| 126 | NPC249342 | –11.6 | –11.6 | –11.6 | 185 | NPC40496 | –10.8 | –11.2 | –11.4 |
| 127 | NPC475561 | –11.5 | –11.6 | –11.6 | 186 | NPC6576 | –9.9 | –10.7 | –11.4 |
| 128 | NPC229160 | –11.0 | –11.5 | –11.6 | 187 | NPC104910 | –8.6 | –11.3 | –11.3 |
| 129 | NPC290534 | –11.7 | –11.9 | –11.6 | 188 | NPC160415 | –10.5 | –11.3 | –11.3 |
| 130 | NPC475138 | –10.4 | –11.4 | –11.6 | 189 | NPC201244 | –11.3 | –11.4 | –11.3 |
| 131 | NPC53520 | –11.1 | –11.9 | –11.6 | 190 | NPC318282 | –10.6 | –11.2 | –11.3 |
| 132 | NPC157311 | –11.0 | –11.5 | –11.6 | 191 | NPC472235 | –11.3 | –11.3 | –11.3 |
| 133 | NPC25316 | –11.4 | –11.5 | –11.6 | 192 | NPC100390 | –11.4 | –11.4 | –11.3 |
| 134 | NPC475367 | –11.3 | –11.5 | –11.6 | 193 | NPC473881 | –9.6 | –10.7 | –11.3 |
| 135 | NPC280385 | –8.7 | –10.8 | –11.5 | 194 | NPC133775 | –11.1 | –10.6 | –11.3 |
| 136 | NPC43213 | –11.4 | –11.5 | –11.5 | 195 | NPC271607 | –10.6 | –11.2 | –11.3 |
| 137 | NPC197357 | –8.7 | –12.2 | –11.5 | 196 | NPC329833 | –11.2 | –11.2 | –11.3 |
| 138 | NPC209058 | –11.5 | –11.5 | –11.5 | 197 | NPC470765 | –10.3 | –11.2 | –11.3 |
| 139 | NPC469312 | –11.4 | –11.5 | –11.5 | 198 | NPC471506 | –11.3 | –11.3 | –11.3 |
| 140 | NPC475196 | –9.8 | –11.1 | –11.5 | 199 | NPC471654 | –11.5 | –11.2 | –11.3 |
| 141 | NPC89923 | –11.2 | –11.4 | –11.5 | 200 | NPC473490 | –9.1 | –10.4 | –11.3 |
| 142 | NPC41129 | –10.0 | –11.4 | –11.5 | 201 | NPC477209 | –11.3 | –11.3 | –11.3 |
| 143 | NPC320801 | –11.2 | –11.3 | –11.5 | 202 | NPC471856 | –11.3 | –11.3 | –11.3 |
| 144 | NPC477292 | –10.3 | –10.8 | –11.5 | 203 | NPC8761 | –10.1 | –11.3 | –11.3 |
| 145 | NPC11242 | –8.9 | –10.9 | –11.5 | 204 | NPC14468 | –10.6 | –11.2 | –11.3 |
| 146 | NPC203486 | –9.1 | –11.3 | –11.5 | 205 | NPC157333 | –10.3 | –11.3 | –11.3 |
| 147 | NPC223301 | –10.1 | –11.2 | –11.5 | 206 | NPC293164 | –11.3 | –11.3 | –11.3 |
| 148 | NPC232130 | –11.5 | –11.5 | –11.5 | 207 | NPC301556 | –9.9 | –10.4 | –11.3 |
| 149 | NPC166492 | –11.2 | –11.2 | –11.5 | 208 | NPC475834 | –11.3 | –11.3 | –11.3 |
| 150 | NPC475165 | –11.5 | –11.5 | –11.5 | 209 | NPC124296 | –8.5 | –10.6 | –11.3 |
| 151 | NPC243981 | –10.3 | –11.3 | –11.5 | 210 | NPC145553 | –11.2 | –11.3 | –11.3 |
| 152 | NPC470015 | –10.7 | –11.2 | –11.5 | 211 | NPC257269 | –8.3 | –11.0 | –11.3 |
| 153 | NPC476958 | –11.5 | –11.5 | –11.5 | 212 | NPC108072 | –9.8 | –10.4 | –11.3 |
| 154 | NPC475424 | –11.3 | –11.5 | –11.5 | 213 | NPC188291 | –11.3 | –11.3 | –11.3 |
| 155 | NPC475913 | –11.5 | –11.5 | –11.5 | 214 | NPC31097 | –11.2 | –11.3 | –11.3 |
| 156 | NPC187456 | –11.3 | –11.1 | –11.5 | 215 | NPC136922 | –9.7 | –10.8 | –11.3 |
| 157 | NPC326264 | –8.7 | –10.5 | –11.5 | 216 | NPC473923 | –10.2 | –11.4 | –11.3 |
| 158 | NPC477959 | –10.3 | –11.3 | –11.4 | 217 | NPC27541 | –9.5 | –11.2 | –11.3 |
| 159 | NPC112274 | –8.6 | –12.4 | –11.4 | 218 | NPC314083 | –8.4 | –10.8 | –11.3 |
| 160 | NPC475288 | –11.4 | –11.4 | –11.4 | 219 | NPC51662 | –9.8 | –11.2 | –11.3 |
| 161 | NPC171148 | –8.1 | –11.3 | –11.4 | 220 | NPC8102 | –11.0 | –11.2 | –11.3 |
| 162 | NPC300183 | –9.7 | –11.4 | –11.4 | 221 | NPC79193 | –11.4 | –11.3 | –11.2 |
| 163 | NPC477208 | –11.4 | –11.4 | –11.4 | 222 | NPC473938 | –11.1 | –11.2 | –11.2 |
| 164 | NPC297617 | –10.5 | –11.4 | –11.4 | 223 | NPC81175 | –11.2 | –10.7 | –11.2 |
| 165 | NPC470753 | –10.4 | –10.4 | –11.4 | 224 | NPC131273 | –10.5 | –11.2 | –11.2 |
| 166 | NPC11266 | –10.5 | –10.6 | –11.4 | 225 | NPC42673 | –11.1 | –11.2 | –11.2 |
| 167 | NPC181298 | –10.1 | –11.4 | –11.4 | 226 | NPC112819 | –10.8 | –11.2 | –11.2 |
| 168 | NPC270498 | –10.4 | –11.0 | –11.4 | 227 | NPC264192 | –11.2 | –11.3 | –11.2 |
| 169 | NPC321496 | –11.1 | –11.4 | –11.4 | 228 | NPC329080 | –8.0 | –11.2 | –11.2 |
| 170 | NPC44805 | –9.5 | –11.4 | –11.4 | 229 | NPC121647 | –10.6 | –10.7 | –11.2 |

**Table S1.** *Continued*.

| No. | Compound Name/Code | Docking Score (kcal/mol) | | | No. | Compound Name/Code | Docking Score (kcal/mol) | | |
|---|---|---|---|---|---|---|---|---|---|
| | | Fast | Moderate | Expensive | | | Fast | Moderate | Expensive |
| 230 | NPC195239 | –11.2 | –11.2 | –11.2 | 289 | NPC139774 | –10.6 | –11.0 | –11.0 |
| 231 | NPC471825 | –11.0 | –11.2 | –11.2 | 290 | NPC152850 | –11.1 | –11.0 | –11.0 |
| 232 | NPC69408 | –11.0 | –11.2 | –11.2 | 291 | NPC259350 | –9.7 | –11.0 | –11.0 |
| 233 | NPC86257 | –11.1 | –11.2 | –11.2 | 292 | NPC473565 | –10.8 | –10.9 | –11.0 |
| 234 | NPC17810 | –11.0 | –11.6 | –11.2 | 293 | NPC118086 | –9.2 | –11.1 | –11.0 |
| 235 | NPC197972 | –8.8 | –11.2 | –11.2 | 294 | NPC40574 | –10.9 | –11.0 | –11.0 |
| 236 | NPC247219 | –10.9 | –11.2 | –11.2 | 295 | NPC469561 | –10.1 | –10.7 | –11.0 |
| 237 | NPC469810 | –10.5 | –10.9 | –11.2 | 296 | NPC475248 | –11.0 | –11.0 | –11.0 |
| 238 | NPC470280 | –11.2 | –10.6 | –11.2 | 297 | NPC476565 | –10.7 | –10.9 | –11.0 |
| 239 | NPC327969 | –10.2 | –11.2 | –11.2 | 298 | NPC477219 | –10.1 | –11.0 | –11.0 |
| 240 | NPC474179 | –11.1 | –11.2 | –11.2 | 299 | NPC101991 | –9.8 | –10.7 | –11.0 |
| 241 | NPC475417 | –11.1 | –11.2 | –11.2 | 300 | NPC160527 | –10.9 | –11.0 | –11.0 |
| 242 | NPC477206 | –10.6 | –11.2 | –11.2 | 301 | NPC473666 | –11.0 | –11.0 | –11.0 |
| 243 | NPC147363 | –10.3 | –11.0 | –11.2 | 302 | NPC230276 | –10.9 | –11.0 | –11.0 |
| 244 | NPC15551 | –11.1 | –11.2 | –11.2 | 303 | NPC314358 | –8.1 | –10.4 | –11.0 |
| 245 | NPC254798 | –8.5 | –11.1 | –11.2 | 304 | NPC471153 | –10.4 | –10.8 | –11.0 |
| 246 | NPC290683 | –10.7 | –10.9 | –11.2 | 305 | NPC223187 | –11.0 | –11.0 | –11.0 |
| 247 | NPC42965 | –11.3 | –10.6 | –11.2 | 306 | NPC227132 | –10.8 | –10.9 | –11.0 |
| 248 | NPC132211 | –11.1 | –11.1 | –11.1 | 307 | NPC470273 | –11.2 | –10.9 | –11.0 |
| 249 | NPC250129 | –11.1 | –11.1 | –11.1 | 308 | NPC474736 | –10.0 | –11.0 | –11.0 |
| 250 | NPC27324 | –10.8 | –11.1 | –11.1 | 309 | NPC149873 | –9.9 | –10.9 | –11.0 |
| 251 | NPC312942 | –10.3 | –10.9 | –11.1 | 310 | NPC243893 | –10.8 | –10.9 | –11.0 |
| 252 | NPC477532 | –10.3 | –11.3 | –11.1 | 311 | NPC310621 | –9.2 | –11.0 | –11.0 |
| 253 | NPC909 | –11.1 | –11.1 | –11.1 | 312 | NPC473493 | –9.2 | –10.7 | –11.0 |
| 254 | NPC161060 | –11.3 | –11.3 | –11.1 | 313 | NPC102810 | –10.1 | –10.9 | –11.0 |
| 255 | NPC270268 | –10.3 | –10.9 | –11.1 | 314 | NPC158347 | –11.0 | –11.0 | –11.0 |
| 256 | NPC469996 | –10.6 | –11.3 | –11.1 | 315 | NPC235885 | –9.7 | –11.0 | –11.0 |
| 257 | NPC475373 | –11.0 | –11.1 | –11.1 | 316 | NPC257175 | –10.6 | –10.9 | –11.0 |
| 258 | NPC214550 | –10.6 | –10.9 | –11.1 | 317 | NPC157828 | –11.0 | –11.0 | –11.0 |
| 259 | NPC473177 | –12.5 | –11.1 | –11.1 | 318 | NPC471394 | –11.0 | –11.0 | –11.0 |
| 260 | NPC473757 | –11.1 | –11.1 | –11.1 | 319 | NPC471601 | –9.3 | –10.8 | –11.0 |
| 261 | NPC83331 | –9.9 | –10.9 | –11.1 | 320 | NPC473593 | –10.9 | –11.0 | –11.0 |
| 262 | NPC242446 | –11.1 | –11.1 | –11.1 | 321 | NPC474923 | –11.0 | –11.0 | –11.0 |
| 263 | NPC471396 | –11.1 | –11.1 | –11.1 | 322 | NPC475436 | –10.9 | –11.0 | –11.0 |
| 264 | NPC477066 | –9.4 | –11.1 | –11.1 | 323 | NPC476963 | –9.9 | –11.0 | –11.0 |
| 265 | NPC102088 | –11.0 | –11.1 | –11.1 | 324 | NPC119493 | –10.3 | –11.0 | –11.0 |
| 266 | NPC71933 | –10.8 | –11.0 | –11.1 | 325 | NPC329731 | –9.6 | –11.1 | –11.0 |
| 267 | NPC97388 | –11.1 | –11.1 | –11.1 | 326 | NPC89317 | –10.7 | –10.9 | –11.0 |
| 268 | NPC158020 | –8.4 | –11.1 | –11.1 | 327 | NPC98457 | –8.4 | –10.8 | –11.0 |
| 269 | NPC160413 | –11.1 | –11.1 | –11.1 | 328 | NPC207243 | –9.8 | –10.7 | –10.9 |
| 270 | NPC171619 | –11.1 | –11.1 | –11.1 | 329 | NPC109512 | –10.9 | –10.9 | –10.9 |
| 271 | NPC263485 | –11.0 | –11.2 | –11.1 | 330 | NPC127790 | –10.9 | –10.9 | –10.9 |
| 272 | NPC26881 | –10.4 | –11.1 | –11.1 | 331 | NPC12890 | –10.9 | –10.9 | –10.9 |
| 273 | NPC475420 | –11.1 | –11.1 | –11.1 | 332 | NPC153792 | –10.9 | –10.9 | –10.9 |
| 274 | NPC79107 | –10.6 | –11.1 | –11.1 | 333 | NPC473115 | –9.3 | –10.6 | –10.9 |
| 275 | NPC10064 | –10.7 | –11.0 | –11.1 | 334 | NPC475602 | –10.9 | –10.9 | –10.9 |
| 276 | NPC116701 | –12.1 | –12.3 | –11.1 | 335 | NPC102253 | –10.8 | –10.8 | –10.9 |
| 277 | NPC180605 | –9.6 | –11.0 | –11.1 | 336 | NPC131693 | –10.4 | –10.7 | –10.9 |
| 278 | NPC181845 | –11.0 | –11.1 | –11.1 | 337 | NPC220974 | –10.8 | –10.9 | –10.9 |
| 279 | NPC315525 | –11.1 | –11.1 | –11.1 | 338 | NPC477362 | –10.8 | –10.9 | –10.9 |
| 280 | NPC120351 | –11.0 | –11.1 | –11.1 | 339 | NPC126002 | –10.8 | –10.9 | –10.9 |
| 281 | NPC323198 | –10.1 | –11.1 | –11.1 | 340 | NPC141970 | –9.8 | –10.8 | –10.9 |
| 282 | NPC473441 | –10.8 | –11.1 | –11.1 | 341 | NPC183570 | –10.1 | –10.9 | –10.9 |
| 283 | NPC476817 | –10.7 | –11.1 | –11.1 | 342 | NPC20302 | –10.7 | –10.9 | –10.9 |
| 284 | NPC24370 | –11.0 | –11.1 | –11.1 | 343 | NPC470277 | –11.6 | –10.9 | –10.9 |
| 285 | NPC73929 | –10.5 | –10.8 | –11.1 | 344 | NPC476599 | –9.7 | –10.9 | –10.9 |
| 286 | NPC20388 | –9.0 | –11.1 | –11.1 | 345 | NPC241477 | –10.9 | –10.9 | –10.9 |
| 287 | NPC233727 | –10.3 | –10.9 | –11.1 | 346 | NPC477291 | –10.2 | –11.0 | –10.9 |
| 288 | NPC477067 | –11.0 | –11.0 | –11.1 | 347 | NPC62792 | –10.3 | –10.7 | –10.9 |

**Table S1.** *Continued*.

| No. | Compound Name/Code | Docking Score (kcal/mol) | | | No. | Compound Name/Code | Docking Score (kcal/mol) | | |
|---|---|---|---|---|---|---|---|---|---|
| | | Fast | Moderate | Expensive | | | Fast | Moderate | Expensive |
| 348 | NPC80650 | –8.0 | –10.9 | –10.9 | 407 | NPC474897 | –10.8 | –10.8 | –10.8 |
| 349 | NPC168319 | –10.9 | –10.9 | –10.9 | 408 | NPC81939 | –8.8 | –10.8 | –10.8 |
| 350 | NPC243594 | –10.8 | –10.9 | –10.9 | 409 | NPC114378 | –10.1 | –10.7 | –10.8 |
| 351 | NPC477391 | –9.2 | –10.5 | –10.9 | 410 | NPC188815 | –9.6 | –10.4 | –10.8 |
| 352 | NPC477901 | –10.3 | –10.9 | –10.9 | 411 | NPC195715 | –10.7 | –10.8 | –10.8 |
| 353 | NPC477914 | –10.9 | –10.9 | –10.9 | 412 | NPC256104 | –10.5 | –10.8 | –10.8 |
| 354 | NPC171007 | –10.9 | –10.9 | –10.9 | 413 | NPC470427 | –10.8 | –10.8 | –10.8 |
| 355 | NPC191382 | –8.8 | –10.7 | –10.9 | 414 | NPC476883 | –10.4 | –10.7 | –10.8 |
| 356 | NPC197447 | –11.2 | –10.9 | –10.9 | 415 | NPC476888 | –10.6 | –10.8 | –10.8 |
| 357 | NPC245055 | –10.8 | –10.9 | –10.9 | 416 | NPC126066 | –10.8 | –10.8 | –10.8 |
| 358 | NPC273467 | –8.3 | –10.9 | –10.9 | 417 | NPC166143 | –10.8 | –10.8 | –10.8 |
| 359 | NPC281134 | –10.2 | –10.8 | –10.9 | 418 | NPC298233 | –10.8 | –10.8 | –10.8 |
| 360 | NPC327095 | –10.6 | –10.8 | –10.9 | 419 | NPC309991 | –10.2 | –10.7 | –10.8 |
| 361 | NPC471403 | –10.8 | –10.9 | –10.9 | 420 | NPC319232 | –10.7 | –10.8 | –10.8 |
| 362 | NPC477903 | –10.6 | –10.5 | –10.9 | 421 | NPC470066 | –10.7 | –10.8 | –10.8 |
| 363 | NPC61411 | –10.9 | –10.9 | –10.9 | 422 | NPC155910 | –9.5 | –10.7 | –10.8 |
| 364 | NPC202866 | –9.4 | –11.0 | –10.9 | 423 | NPC180204 | –10.5 | –10.8 | –10.8 |
| 365 | NPC475369 | –10.5 | –10.9 | –10.9 | 424 | NPC6206 | –10.0 | –10.7 | –10.8 |
| 366 | NPC475605 | –10.0 | –10.8 | –10.9 | 425 | NPC12103 | –9.8 | –10.6 | –10.8 |
| 367 | NPC74969 | –10.8 | –10.9 | –10.9 | 426 | NPC202198 | –10.2 | –10.4 | –10.8 |
| 368 | NPC124358 | –8.6 | –10.5 | –10.9 | 427 | NPC227275 | –8.9 | –10.7 | –10.8 |
| 369 | NPC272549 | –10.8 | –10.9 | –10.9 | 428 | NPC29695 | –10.3 | –10.6 | –10.8 |
| 370 | NPC471541 | –11.7 | –10.7 | –10.9 | 429 | NPC4574 | –10.0 | –10.7 | –10.8 |
| 371 | NPC472361 | –12.5 | –11.7 | –10.9 | 430 | NPC470309 | –9.9 | –10.7 | –10.8 |
| 372 | NPC6215 | –10.9 | –10.9 | –10.9 | 431 | NPC470882 | –10.8 | –10.8 | –10.8 |
| 373 | NPC16657 | –10.4 | –10.7 | –10.9 | 432 | NPC473178 | –11.5 | –10.7 | –10.8 |
| 374 | NPC471617 | –9.6 | –10.8 | –10.9 | 433 | NPC474315 | –10.6 | –10.7 | –10.8 |
| 375 | NPC476257 | –11.0 | –10.7 | –10.9 | 434 | NPC476491 | –10.2 | –10.7 | –10.8 |
| 376 | NPC476890 | –10.6 | –10.8 | –10.9 | 435 | NPC132771 | –10.4 | –10.8 | –10.7 |
| 377 | NPC184933 | –10.8 | –11.0 | –10.8 | 436 | NPC138974 | –9.9 | –10.7 | –10.7 |
| 378 | NPC61382 | –11.2 | –11.0 | –10.8 | 437 | NPC224557 | –10.2 | –10.7 | –10.7 |
| 379 | NPC183580 | –10.8 | –10.8 | –10.8 | 438 | NPC253268 | –10.6 | –10.8 | –10.7 |
| 380 | NPC242728 | –11.0 | –10.3 | –10.8 | 439 | NPC266374 | –8.9 | –10.8 | –10.7 |
| 381 | NPC246904 | –10.7 | –10.8 | –10.8 | 440 | NPC277351 | –10.8 | –10.8 | –10.7 |
| 382 | NPC43353 | –10.6 | –10.8 | –10.8 | 441 | NPC292775 | –10.7 | –10.7 | –10.7 |
| 383 | NPC198893 | –8.2 | –11.9 | –10.8 | 442 | NPC292793 | –10.6 | –10.7 | –10.7 |
| 384 | NPC253456 | –10.9 | –10.8 | –10.8 | 443 | NPC474571 | –10.8 | –10.7 | –10.7 |
| 385 | NPC286528 | –10.0 | –10.8 | –10.8 | 444 | NPC476750 | –9.6 | –10.7 | –10.7 |
| 386 | NPC33064 | –10.1 | –10.8 | –10.8 | 445 | NPC476889 | –10.5 | –10.7 | –10.7 |
| 387 | NPC469722 | –10.8 | –10.8 | –10.8 | 446 | NPC291634 | –10.3 | –10.5 | –10.7 |
| 388 | NPC475654 | –10.8 | –10.8 | –10.8 | 447 | NPC3381 | –10.2 | –10.6 | –10.7 |
| 389 | NPC101067 | –10.8 | –10.8 | –10.8 | 448 | NPC470960 | –10.7 | –10.7 | –10.7 |
| 390 | NPC188239 | –10.8 | –10.8 | –10.8 | 449 | NPC471994 | –10.7 | –10.7 | –10.7 |
| 391 | NPC325740 | –10.5 | –11.0 | –10.8 | 450 | NPC478108 | –9.7 | –10.4 | –10.7 |
| 392 | NPC471432 | –10.6 | –10.7 | –10.8 | 451 | NPC191489 | –9.9 | –10.7 | –10.7 |
| 393 | NPC473851 | –9.9 | –10.4 | –10.8 | 452 | NPC204652 | –10.2 | –10.6 | –10.7 |
| 394 | NPC120994 | –9.8 | –10.7 | –10.8 | 453 | NPC22248 | –10.7 | –11.0 | –10.7 |
| 395 | NPC156298 | –9.5 | –12.2 | –10.8 | 454 | NPC271387 | –10.0 | –10.7 | –10.7 |
| 396 | NPC175477 | –10.8 | –10.8 | –10.8 | 455 | NPC280566 | –11.4 | –10.7 | –10.7 |
| 397 | NPC223236 | –10.5 | –10.8 | –10.8 | 456 | NPC96759 | –10.7 | –10.7 | –10.7 |
| 398 | NPC287063 | –9.8 | –10.8 | –10.8 | 457 | NPC233273 | –10.1 | –10.7 | –10.7 |
| 399 | NPC312536 | –10.7 | –10.8 | –10.8 | 458 | NPC320475 | –10.5 | –10.7 | –10.7 |
| 400 | NPC328287 | –9.5 | –10.8 | –10.8 | 459 | NPC324001 | –11.0 | –11.0 | –10.7 |
| 401 | NPC43747 | –9.8 | –10.5 | –10.8 | 460 | NPC476879 | –9.4 | –10.6 | –10.7 |
| 402 | NPC471135 | –8.7 | –10.8 | –10.8 | 461 | NPC476960 | –9.7 | –10.7 | –10.7 |
| 403 | NPC477392 | –8.2 | –10.5 | –10.8 | 462 | NPC132386 | –10.7 | –10.7 | –10.7 |
| 404 | NPC88326 | –10.8 | –11.3 | –10.8 | 463 | NPC290261 | –9.0 | –10.5 | –10.7 |
| 405 | NPC137657 | –10.8 | –10.8 | –10.8 | 464 | NPC315499 | –10.9 | –10.7 | –10.7 |
| 406 | NPC329761 | –9.1 | –10.7 | –10.8 | 465 | NPC317590 | –10.3 | –10.6 | –10.7 |

**Table S1.** *Continued*.

| No. | Compound Name/Code | Docking Score (kcal/mol) | | | No. | Compound Name/Code | Docking Score (kcal/mol) | | |
|---|---|---|---|---|---|---|---|---|---|
| | | Fast | Moderate | Expensive | | | Fast | Moderate | Expensive |
| 466 | NPC96016 | –9.3 | –10.5 | –10.7 | 525 | NPC471963 | –8.2 | –10.4 | –10.6 |
| 467 | NPC157817 | –10.7 | –10.8 | –10.7 | 526 | NPC472206 | –10.6 | –10.6 | –10.6 |
| 468 | NPC211845 | –10.7 | –10.7 | –10.7 | 527 | NPC472238 | –10.6 | –10.6 | –10.6 |
| 469 | NPC241935 | –10.7 | –10.7 | –10.7 | 528 | NPC477101 | –9.0 | –10.6 | –10.6 |
| 470 | NPC2497 | –10.7 | –10.7 | –10.7 | 529 | NPC478060 | –10.2 | –10.6 | –10.6 |
| 471 | NPC289776 | –9.1 | –10.7 | –10.7 | 530 | NPC58052 | –9.5 | –10.6 | –10.6 |
| 472 | NPC298647 | –10.4 | –10.7 | –10.7 | 531 | NPC159338 | –10.5 | –10.6 | –10.6 |
| 473 | NPC322353 | –10.5 | –10.6 | –10.7 | 532 | NPC178025 | –10.4 | –10.6 | –10.6 |
| 474 | NPC478261 | –10.7 | –10.7 | –10.7 | 533 | NPC313867 | –8.8 | –10.5 | –10.6 |
| 475 | NPC79921 | –9.2 | –10.7 | –10.7 | 534 | NPC470750 | –10.4 | –10.6 | –10.6 |
| 476 | NPC85173 | –10.7 | –10.7 | –10.7 | 535 | NPC472231 | –10.6 | –10.6 | –10.6 |
| 477 | NPC144202 | –10.3 | –10.6 | –10.7 | 536 | NPC474190 | –10.2 | –10.9 | –10.6 |
| 478 | NPC199259 | –10.7 | –10.7 | –10.7 | 537 | NPC475244 | –10.6 | –10.6 | –10.6 |
| 479 | NPC254581 | –10.7 | –10.7 | –10.7 | 538 | NPC476077 | –10.6 | –11.2 | –10.6 |
| 480 | NPC322982 | –10.2 | –10.7 | –10.7 | 539 | NPC476126 | –10.6 | –10.6 | –10.6 |
| 481 | NPC469824 | –9.2 | –10.7 | –10.7 | 540 | NPC476878 | –10.2 | –10.6 | –10.6 |
| 482 | NPC476097 | –10.7 | –10.7 | –10.7 | 541 | NPC70155 | –10.6 | –10.6 | –10.6 |
| 483 | NPC57690 | –10.6 | –10.7 | –10.7 | 542 | NPC13494 | –9.6 | –10.4 | –10.6 |
| 484 | NPC227583 | –9.0 | –10.7 | –10.7 | 543 | NPC187255 | –10.3 | –10.5 | –10.6 |
| 485 | NPC241050 | –9.6 | –11.0 | –10.7 | 544 | NPC19013 | –10.6 | –10.6 | –10.6 |
| 486 | NPC268676 | –10.5 | –10.7 | –10.7 | 545 | NPC243065 | –10.6 | –10.6 | –10.6 |
| 487 | NPC469382 | –10.2 | –10.6 | –10.7 | 546 | NPC273199 | –10.6 | –10.6 | –10.6 |
| 488 | NPC470623 | –9.7 | –10.8 | –10.7 | 547 | NPC295676 | –10.6 | –10.6 | –10.6 |
| 489 | NPC472225 | –10.7 | –10.7 | –10.7 | 548 | NPC472863 | –8.6 | –10.5 | –10.6 |
| 490 | NPC124029 | –9.1 | –10.4 | –10.7 | 549 | NPC473535 | –9.6 | –10.6 | –10.6 |
| 491 | NPC267965 | –10.7 | –10.7 | –10.7 | 550 | NPC475230 | –10.5 | –10.6 | –10.6 |
| 492 | NPC284102 | –10.7 | –10.7 | –10.7 | 551 | NPC477494 | –8.5 | –10.6 | –10.6 |
| 493 | NPC289905 | –10.5 | –10.6 | –10.7 | 552 | NPC103782 | –9.2 | –10.6 | –10.6 |
| 494 | NPC473006 | –10.0 | –10.7 | –10.7 | 553 | NPC209174 | –10.6 | –10.6 | –10.6 |
| 495 | NPC475597 | –10.7 | –10.7 | –10.7 | 554 | NPC235557 | –10.1 | –10.5 | –10.6 |
| 496 | NPC91125 | –8.4 | –10.5 | –10.7 | 555 | NPC240042 | –8.0 | –10.5 | –10.6 |
| 497 | NPC141957 | –10.0 | –10.5 | –10.7 | 556 | NPC268530 | –10.5 | –10.6 | –10.6 |
| 498 | NPC147635 | –10.1 | –10.6 | –10.7 | 557 | NPC32064 | –10.6 | –10.6 | –10.6 |
| 499 | NPC252338 | –10.2 | –10.4 | –10.7 | 558 | NPC477899 | –10.5 | –11.4 | –10.6 |
| 500 | NPC270511 | –10.2 | –10.6 | –10.7 | 559 | NPC60150 | –10.6 | –10.6 | –10.6 |
| 501 | NPC312630 | –10.6 | –10.7 | –10.7 | 560 | NPC242774 | –10.3 | –10.6 | –10.6 |
| 502 | NPC323752 | –10.5 | –10.7 | –10.7 | 561 | NPC286786 | –10.5 | –10.6 | –10.6 |
| 503 | NPC37116 | –10.6 | –10.7 | –10.7 | 562 | NPC42670 | –10.6 | –10.6 | –10.6 |
| 504 | NPC477556 | –10.4 | –10.6 | –10.7 | 563 | NPC470418 | –9.2 | –10.6 | –10.6 |
| 505 | NPC51653 | –9.1 | –10.6 | –10.7 | 564 | NPC475988 | –10.6 | –10.6 | –10.6 |
| 506 | NPC58029 | –10.7 | –10.6 | –10.7 | 565 | NPC135043 | –10.6 | –10.6 | –10.6 |
| 507 | NPC262077 | –9.1 | –10.6 | –10.6 | 566 | NPC174122 | –8.4 | –10.5 | –10.6 |
| 508 | NPC45576 | –10.6 | –10.6 | –10.6 | 567 | NPC241636 | –10.6 | –10.6 | –10.6 |
| 509 | NPC470874 | –10.7 | –10.6 | –10.6 | 568 | NPC30222 | –9.4 | –10.5 | –10.6 |
| 510 | NPC472673 | –9.1 | –10.6 | –10.6 | 569 | NPC470274 | –11.0 | –10.5 | –10.6 |
| 511 | NPC105073 | –10.3 | –10.6 | –10.6 | 570 | NPC474106 | –10.5 | –10.6 | –10.6 |
| 512 | NPC118033 | –10.2 | –10.6 | –10.6 | 571 | NPC474107 | –10.3 | –10.6 | –10.6 |
| 513 | NPC23621 | –10.6 | –10.6 | –10.6 | 572 | NPC475718 | –8.9 | –10.6 | –10.6 |
| 514 | NPC74639 | –10.7 | –10.6 | –10.6 | 573 | NPC49911 | –10.6 | –10.6 | –10.6 |
| 515 | NPC78473 | –10.4 | –10.6 | –10.6 | 574 | NPC116742 | –9.3 | –10.6 | –10.6 |
| 516 | NPC96131 | –11.3 | –10.4 | –10.6 | 575 | NPC33949 | –10.5 | –10.5 | –10.6 |
| 517 | NPC15215 | –10.6 | –10.6 | –10.6 | 576 | NPC472926 | –9.9 | –10.4 | –10.6 |
| 518 | NPC155974 | –10.4 | –10.6 | –10.6 | 577 | NPC473279 | –10.6 | –10.6 | –10.6 |
| 519 | NPC176246 | –8.6 | –12.0 | –10.6 | 578 | NPC476216 | –10.6 | –10.5 | –10.6 |
| 520 | NPC202705 | –10.4 | –10.6 | –10.6 | 579 | NPC48305 | –10.6 | –10.6 | –10.6 |
| 521 | NPC27615 | –10.6 | –10.7 | –10.6 | 580 | NPC5292 | –6.9 | –10.5 | –10.6 |
| 522 | NPC283343 | –10.6 | –10.6 | –10.6 | 581 | NPC87393 | –10.2 | –10.5 | –10.6 |
| 523 | NPC300557 | –10.3 | –10.5 | –10.6 | 582 | NPC88668 | –8.5 | –10.4 | –10.6 |
| 524 | NPC41376 | –9.9 | –10.6 | –10.6 | 583 | NPC150977 | –9.9 | –10.5 | –10.6 |

**Table S1.** *Continued*.

| No. | Compound Name/Code | Docking Score (kcal/mol) | | | No. | Compound Name/Code | Docking Score (kcal/mol) | | |
|---|---|---|---|---|---|---|---|---|---|
| | | Fast | Moderate | Expensive | | | Fast | Moderate | Expensive |
| 584 | NPC20734 | –10.0 | –10.5 | –10.6 | 643 | NPC472233 | –10.5 | –10.5 | –10.5 |
| 585 | NPC254146 | –10.6 | –10.6 | –10.6 | 644 | NPC475296 | –8.1 | –10.5 | –10.5 |
| 586 | NPC312660 | –10.5 | –10.6 | –10.6 | 645 | NPC93744 | –10.4 | –10.5 | –10.5 |
| 587 | NPC313921 | –10.6 | –10.6 | –10.6 | 646 | NPC190442 | –10.5 | –10.5 | –10.5 |
| 588 | NPC315848 | –8.6 | –10.4 | –10.6 | 647 | NPC225821 | –10.4 | –10.5 | –10.5 |
| 589 | NPC32017 | –10.2 | –10.6 | –10.6 | 648 | NPC240604 | –10.5 | –10.5 | –10.5 |
| 590 | NPC469452 | –10.5 | –10.7 | –10.6 | 649 | NPC266955 | –10.1 | –10.4 | –10.5 |
| 591 | NPC473467 | –10.5 | –10.6 | –10.6 | 650 | NPC4309 | –10.3 | –10.5 | –10.5 |
| 592 | NPC474790 | –10.3 | –10.5 | –10.6 | 651 | NPC470076 | –10.3 | –10.5 | –10.5 |
| 593 | NPC131405 | –11.2 | –11.8 | –10.5 | 652 | NPC472226 | –10.5 | –10.5 | –10.5 |
| 594 | NPC208994 | –10.0 | –10.3 | –10.5 | 653 | NPC473429 | –10.2 | –10.5 | –10.5 |
| 595 | NPC218301 | –10.5 | –10.5 | –10.5 | 654 | NPC475719 | –10.4 | –10.5 | –10.5 |
| 596 | NPC229976 | –10.5 | –10.5 | –10.5 | 655 | NPC477042 | –10.5 | –10.5 | –10.5 |
| 597 | NPC237182 | –10.0 | –10.5 | –10.5 | 656 | NPC477715 | –9.9 | –10.4 | –10.5 |
| 598 | NPC315974 | –10.0 | –10.5 | –10.5 | 657 | NPC477857 | –9.5 | –10.4 | –10.5 |
| 599 | NPC316930 | –10.1 | –10.4 | –10.5 | 658 | NPC282905 | –10.5 | –10.5 | –10.5 |
| 600 | NPC32715 | –9.8 | –10.4 | –10.5 | 659 | NPC40749 | –8.3 | –10.5 | –10.5 |
| 601 | NPC470873 | –10.5 | –10.5 | –10.5 | 660 | NPC470068 | –10.3 | –10.4 | –10.5 |
| 602 | NPC475548 | –10.5 | –10.5 | –10.5 | 661 | NPC477099 | –9.4 | –10.4 | –10.5 |
| 603 | NPC67569 | –9.7 | –10.5 | –10.5 | 662 | NPC477557 | –10.5 | –10.5 | –10.5 |
| 604 | NPC86346 | –10.3 | –10.5 | –10.5 | 663 | NPC51247 | –10.3 | –10.5 | –10.5 |
| 605 | NPC101886 | –10.5 | –10.5 | –10.5 | 664 | NPC90822 | –10.3 | –10.4 | –10.5 |
| 606 | NPC122056 | –10.5 | –10.5 | –10.5 | 665 | NPC163693 | –10.4 | –10.4 | –10.5 |
| 607 | NPC13743 | –9.7 | –10.5 | –10.5 | 666 | NPC176840 | –10.4 | –10.5 | –10.5 |
| 608 | NPC473573 | –10.4 | –10.5 | –10.5 | 667 | NPC18982 | –10.4 | –10.4 | –10.5 |
| 609 | NPC478036 | –10.1 | –10.5 | –10.5 | 668 | NPC247563 | –10.3 | –10.4 | –10.5 |
| 610 | NPC8374 | –10.5 | –10.5 | –10.5 | 669 | NPC255082 | –10.2 | –10.5 | –10.5 |
| 611 | NPC133506 | –10.5 | –10.5 | –10.5 | 670 | NPC255309 | –10.4 | –10.4 | –10.5 |
| 612 | NPC262083 | –10.5 | –10.5 | –10.5 | 671 | NPC27918 | –10.5 | –10.5 | –10.5 |
| 613 | NPC264875 | –9.3 | –10.6 | –10.5 | 672 | NPC298072 | –10.0 | –10.4 | –10.5 |
| 614 | NPC470419 | –10.5 | –10.5 | –10.5 | 673 | NPC310981 | –10.6 | –10.5 | –10.5 |
| 615 | NPC475027 | –10.4 | –10.5 | –10.5 | 674 | NPC34177 | –10.5 | –10.5 | –10.5 |
| 616 | NPC475151 | –9.9 | –10.5 | –10.5 | 675 | NPC470954 | –10.5 | –10.5 | –10.5 |
| 617 | NPC17274 | –9.5 | –10.5 | –10.5 | 676 | NPC185530 | –10.4 | –10.5 | –10.5 |
| 618 | NPC266513 | –9.6 | –11.6 | –10.5 | 677 | NPC234548 | –12.0 | –10.4 | –10.5 |
| 619 | NPC285184 | –10.4 | –10.5 | –10.5 | 678 | NPC471404 | –10.3 | –10.5 | –10.5 |
| 620 | NPC290746 | –9.6 | –10.6 | –10.5 | 679 | NPC473734 | –8.6 | –10.4 | –10.5 |
| 621 | NPC41318 | –10.4 | –10.5 | –10.5 | 680 | NPC475274 | –10.3 | –10.4 | –10.5 |
| 622 | NPC97078 | –10.0 | –10.5 | –10.5 | 681 | NPC476080 | –8.8 | –10.6 | –10.5 |
| 623 | NPC114441 | –8.3 | –10.3 | –10.5 | 682 | NPC477363 | –10.3 | –10.5 | –10.5 |
| 624 | NPC126128 | –10.5 | –10.5 | –10.5 | 683 | NPC153700 | –9.5 | –10.4 | –10.4 |
| 625 | NPC138775 | –9.6 | –10.4 | –10.5 | 684 | NPC174024 | –10.1 | –10.8 | –10.4 |
| 626 | NPC139783 | –10.5 | –10.5 | –10.5 | 685 | NPC245410 | –10.4 | –10.4 | –10.4 |
| 627 | NPC178541 | –10.5 | –10.5 | –10.5 | 686 | NPC267926 | –10.4 | –10.4 | –10.4 |
| 628 | NPC289383 | –10.1 | –11.6 | –10.5 | 687 | NPC27518 | –10.3 | –10.4 | –10.4 |
| 629 | NPC293038 | –10.2 | –10.5 | –10.5 | 688 | NPC307396 | –10.4 | –10.4 | –10.4 |
| 630 | NPC306799 | –10.1 | –10.4 | –10.5 | 689 | NPC40918 | –10.4 | –10.4 | –10.4 |
| 631 | NPC473173 | –10.4 | –10.5 | –10.5 | 690 | NPC471113 | –10.4 | –10.4 | –10.4 |
| 632 | NPC474370 | –9.7 | –10.5 | –10.5 | 691 | NPC478258 | –10.5 | –10.5 | –10.4 |
| 633 | NPC476091 | –10.3 | –10.5 | –10.5 | 692 | NPC49964 | –10.2 | –10.4 | –10.4 |
| 634 | NPC4910 | –10.4 | –10.4 | –10.5 | 693 | NPC99411 | –9.7 | –10.3 | –10.4 |
| 635 | NPC108476 | –10.5 | –10.5 | –10.5 | 694 | NPC151292 | –8.1 | –10.4 | –10.4 |
| 636 | NPC126815 | –10.4 | –10.5 | –10.5 | 695 | NPC278540 | –10.3 | –10.4 | –10.4 |
| 637 | NPC242068 | –10.4 | –10.5 | –10.5 | 696 | NPC39431 | –10.4 | –10.4 | –10.4 |
| 638 | NPC244606 | –10.5 | –10.5 | –10.5 | 697 | NPC469731 | –10.4 | –10.4 | –10.4 |
| 639 | NPC284162 | –10.5 | –10.5 | –10.5 | 698 | NPC470053 | –9.4 | –10.4 | –10.4 |
| 640 | NPC470126 | –9.6 | –10.5 | –10.5 | 699 | NPC475238 | –10.4 | –10.4 | –10.4 |
| 641 | NPC470223 | –9.2 | –10.5 | –10.5 | 700 | NPC56618 | –9.7 | –10.4 | –10.4 |
| 642 | NPC472229 | –10.5 | –10.5 | –10.5 | 701 | NPC93662 | –10.4 | –10.4 | –10.4 |

**Table S1.** *Continued*.

| No. | Compound Name/Code | Docking Score (kcal/mol) | | | No. | Compound Name/Code | Docking Score (kcal/mol) | | |
|---|---|---|---|---|---|---|---|---|---|
| | | Fast | Moderate | Expensive | | | Fast | Moderate | Expensive |
| 702 | NPC117027 | –10.4 | –10.4 | –10.4 | 761 | NPC50615 | –9.5 | –11.4 | –10.3 |
| 703 | NPC177064 | –10.4 | –10.4 | –10.4 | 762 | NPC185665 | –10.3 | –10.3 | –10.3 |
| 704 | NPC198621 | –9.0 | –10.3 | –10.4 | 763 | NPC475253 | –9.7 | –10.4 | –10.3 |
| 705 | NPC209509 | –10.1 | –10.4 | –10.4 | 764 | NPC74727 | –10.3 | –10.4 | –10.3 |
| 706 | NPC273623 | –10.3 | –10.4 | –10.4 | 765 | NPC162033 | –10.5 | –11.1 | –10.3 |
| 707 | NPC293044 | –10.4 | –10.4 | –10.4 | 766 | NPC235364 | –9.7 | –10.3 | –10.3 |
| 708 | NPC470425 | –10.4 | –10.4 | –10.4 | 767 | NPC289086 | –9.8 | –10.4 | –10.3 |
| 709 | NPC471720 | –10.4 | –10.4 | –10.4 | 768 | NPC141053 | –10.0 | –10.4 | –10.3 |
| 710 | NPC473310 | –10.2 | –10.4 | –10.4 | 769 | NPC476830 | –9.9 | –10.7 | –10.3 |
| 711 | NPC474448 | –10.5 | –10.4 | –10.4 | 770 | NPC270586 | –9.1 | –10.4 | –10.2 |
| 712 | NPC93416 | –10.5 | –10.5 | –10.4 | 771 | NPC320026 | –10.5 | –10.7 | –10.2 |
| 713 | NPC110882 | –10.2 | –10.4 | –10.4 | 772 | NPC159526 | –9.6 | –10.4 | –10.2 |
| 714 | NPC194427 | –10.3 | –10.4 | –10.4 | 773 | NPC474564 | –11.2 | –12.0 | –10.2 |
| 715 | NPC206592 | –10.4 | –10.4 | –10.4 | 774 | NPC208189 | –10.6 | –10.8 | –10.1 |
| 716 | NPC209851 | –8.6 | –10.4 | –10.4 | 775 | NPC212768 | –10.2 | –10.5 | –10.1 |
| 717 | NPC251936 | –10.4 | –10.4 | –10.4 | 776 | NPC475540 | –9.0 | –10.6 | –10.1 |
| 718 | NPC305501 | –10.2 | –10.4 | –10.4 | 777 | NPC107674 | –10.1 | –10.3 | –10.1 |
| 719 | NPC34963 | –10.4 | –10.4 | –10.4 | 778 | NPC473085 | –9.1 | –10.4 | –10.1 |
| 720 | NPC472234 | –10.4 | –10.4 | –10.4 | 779 | NPC235824 | –8.6 | –10.5 | –10.1 |
| 721 | NPC472294 | –8.9 | –10.4 | –10.4 | 780 | NPC48599 | –9.3 | –10.5 | –10.0 |
| 722 | NPC473591 | –10.0 | –10.3 | –10.4 | 781 | NPC470312 | –8.5 | –10.6 | –10.0 |
| 723 | NPC477906 | –9.0 | –10.4 | –10.4 | 782 | NPC470892 | –9.0 | –10.4 | –10.0 |
| 724 | NPC120917 | –10.3 | –10.3 | –10.4 | 783 | NPC119481 | –10.5 | –11.2 | –10.0 |
| 725 | NPC151030 | –8.2 | –10.4 | –10.4 | 784 | NPC137414 | –9.0 | –10.4 | –10.0 |
| 726 | NPC168511 | –10.4 | –10.4 | –10.4 | 785 | NPC471049 | –9.5 | –10.5 | –9.9 |
| 727 | NPC274448 | –10.2 | –10.4 | –10.4 | 786 | NPC473220 | –9.6 | –10.9 | –9.9 |
| 728 | NPC310572 | –9.8 | –10.4 | –10.4 | 787 | NPC478127 | –9.6 | –10.7 | –9.9 |
| 729 | NPC43716 | –8.7 | –10.3 | –10.4 | 788 | NPC136948 | –10.1 | –10.4 | –9.9 |
| 730 | NPC469720 | –10.4 | –10.4 | –10.4 | 789 | NPC302392 | –10.3 | –10.3 | –9.9 |
| 731 | NPC472223 | –10.4 | –10.4 | –10.4 | 790 | NPC475651 | –11.0 | –11.5 | –9.9 |
| 732 | NPC474870 | –10.4 | –10.4 | –10.4 | 791 | NPC3952 | –10.7 | –10.7 | –9.8 |
| 733 | NPC48339 | –9.5 | –10.7 | –10.4 | 792 | NPC85316 | –9.7 | –11.5 | –9.8 |
| 734 | NPC153734 | –10.4 | –10.4 | –10.4 | 793 | NPC475249 | –8.1 | –10.9 | –9.8 |
| 735 | NPC190401 | –9.8 | –10.4 | –10.4 | 794 | NPC202898 | –10.9 | –12.2 | –9.8 |
| 736 | NPC472695 | –8.8 | –10.4 | –10.4 | 795 | NPC477468 | –9.7 | –10.6 | –9.7 |
| 737 | NPC108840 | –10.4 | –10.4 | –10.4 | 796 | NPC471777 | –9.0 | –11.2 | –9.7 |
| 738 | NPC255401 | –10.4 | –10.4 | –10.4 | 797 | NPC470275 | –10.5 | –10.8 | –9.7 |
| 739 | NPC275575 | –9.8 | –10.4 | –10.4 | 798 | NPC25736 | –9.9 | –10.6 | –9.7 |
| 740 | NPC312678 | –9.8 | –11.0 | –10.4 | 799 | NPC308262 | –9.8 | –11.1 | –9.6 |
| 741 | NPC469740 | –10.5 | –10.4 | –10.4 | 800 | NPC328928 | –9.3 | –10.9 | –9.6 |
| 742 | NPC65700 | –10.8 | –10.4 | –10.4 | 801 | NPC471582 | –10.6 | –11.0 | –9.6 |
| 743 | NPC289486 | –10.3 | –10.4 | –10.4 | 802 | NPC475536 | –9.5 | –10.8 | –9.6 |
| 744 | NPC41679 | –10.4 | –10.4 | –10.4 | 803 | NPC93685 | –10.9 | –11.1 | –9.6 |
| 745 | NPC469394 | –10.0 | –10.4 | –10.4 | 804 | NPC38350 | –10.3 | –10.4 | –9.6 |
| 746 | NPC470036 | –10.3 | –10.4 | –10.4 | 805 | NPC469317 | –8.3 | –10.5 | –9.5 |
| 747 | NPC472230 | –10.4 | –10.4 | –10.4 | 806 | NPC99088 | –10.9 | –11.4 | –9.5 |
| 748 | NPC473680 | –10.2 | –10.3 | –10.4 | 807 | NPC304322 | –11.0 | –11.6 | –9.5 |
| 749 | NPC475862 | –10.4 | –10.4 | –10.4 | 808 | NPC477902 | –9.4 | –10.5 | –9.5 |
| 750 | NPC18536 | –10.3 | –10.4 | –10.4 | 809 | NPC69273 | –8.5 | –10.9 | –9.5 |
| 751 | NPC204491 | –8.3 | –10.3 | –10.4 | 810 | NPC102843 | –9.5 | –12.1 | –9.4 |
| 752 | NPC292233 | –9.3 | –10.3 | –10.4 | 811 | NPC471980 | –9.6 | –11.2 | –9.4 |
| 753 | NPC35164 | –10.3 | –10.4 | –10.4 | 812 | NPC473749 | –8.2 | –10.8 | –9.4 |
| 754 | NPC470037 | –10.3 | –10.3 | –10.4 | 813 | NPC146563 | –8.3 | –10.5 | –9.4 |
| 755 | NPC57079 | –10.1 | –10.3 | –10.4 | 814 | NPC97884 | –8.9 | –10.8 | –9.4 |
| 756 | NPC104387 | –10.4 | –10.4 | –10.3 | 815 | NPC269095 | –10.8 | –10.8 | –9.4 |
| 757 | NPC249817 | –10.2 | –10.3 | –10.3 | 816 | NPC177834 | –9.1 | –10.7 | –9.3 |
| 758 | NPC6274 | –10.3 | –10.4 | –10.3 | 817 | NPC235841 | –8.1 | –10.4 | –9.2 |
| 759 | NPC472237 | –10.3 | –10.3 | –10.3 | 818 | NPC323074 | –9.3 | –10.7 | –9.2 |
| 760 | NPC47386 | –10.1 | –10.4 | –10.3 | 819 | NPC10005 | –9.1 | –10.4 | –9.1 |

**Table S1.** *Continued*.

| No. | Compound Name/Code | Docking Score (kcal/mol) | | | No. | Compound Name/Code | Docking Score (kcal/mol) | | |
|---|---|---|---|---|---|---|---|---|---|
| | | Fast | Moderate | Expensive | | | Fast | Moderate | Expensive |
| 820 | NPC271494 | –8.5 | –10.6 | –9.1 | 844 | NPC473160 | –10.4 | –10.3 | –6.6 |
| 821 | NPC476784 | –11.0 | –10.4 | –9.1 | 845 | NPC473596 | –9.5 | –10.3 | –6.6 |
| 822 | NPC470504 | –9.6 | –10.4 | –9.1 | 846 | NPC474976 | –10.3 | –10.3 | –6.6 |
| 823 | NPC102725 | –10.1 | –10.4 | –9.0 | 847 | NPC475025 | –10.2 | –10.3 | –6.6 |
| 824 | NPC472215 | –8.5 | –10.5 | –9.0 | 848 | NPC477334 | –9.3 | –10.3 | –6.6 |
| 825 | NPC473890 | –9.2 | –10.7 | –9.0 | 849 | NPC63023 | –9.9 | –10.3 | –6.6 |
| 826 | NPC473020 | –8.7 | –10.4 | –8.9 | 850 | NPC11847 | –9.9 | –10.3 | –6.6 |
| 827 | NPC45943 | –9.0 | –10.9 | –8.9 | 851 | NPC122116 | –10.3 | –10.3 | –6.6 |
| 828 | NPC229962 | –8.9 | –10.4 | –8.8 | 852 | NPC133625 | –8.3 | –10.3 | –6.5 |
| 829 | NPC40716 | –10.3 | –10.3 | –8.8 | 853 | NPC16350 | –8.2 | –10.3 | –6.5 |
| 830 | NPC278272 | –9.4 | –11.3 | –8.7 | 854 | NPC473160 | –10.4 | –10.3 | –6.5 |
| 831 | NPC115601 | –9.3 | –11.1 | –8.7 | 855 | NPC473596 | –9.5 | –10.3 | –6.5 |
| 832 | NPC116024 | –9.3 | –10.6 | –8.6 | 856 | NPC474976 | –10.3 | –10.3 | –6.5 |
| 833 | NPC220293 | –10.1 | –10.8 | –8.6 | 857 | NPC475025 | –10.2 | –10.3 | –6.5 |
| 834 | NPC160084 | –10.5 | –11.5 | –8.5 | 858 | NPC477334 | –9.3 | –10.3 | –6.5 |
| 835 | NPC273798 | –9.1 | –10.5 | –8.5 | 859 | NPC63023 | –9.9 | –10.3 | –6.4 |
| 836 | NPC286809 | –11.3 | –12.1 | –8.2 | 860 | NPC11847 | –9.9 | –10.3 | –6.4 |
| 837 | NPC165234 | –10.6 | –12.3 | –7.9 | 861 | NPC122116 | –10.3 | –10.3 | –6.4 |
| 838 | NPC318581 | –9.1 | –10.8 | –7.8 | 862 | NPC133625 | –8.3 | –10.3 | –6.4 |
| 839 | NPC114287 | –10.7 | –11.1 | –7.6 | 863 | NPC16350 | –10.3 | –10.3 | –6.4 |
| 840 | NPC117478 | –8.1 | –10.4 | –7.4 | 864 | NPC190204 | –8.2 | –10.3 | –6.4 |
| 841 | NPC21691 | –8.4 | –11.6 | –6.9 | 865 | NPC200589 | –9.1 | –10.3 | –6.4 |
| 842 | NPC327699 | –10.2 | –11.3 | –6.8 | 866 | NPC201634 | –10.3 | –10.3 | –6.4 |
| 843 | NPC477074 | –8.7 | –10.6 | –6.6 | | | | | |

[a]Data ranked based on the Expensive docking score.

**Table S2.** Estimated docking scores and MM-GBSA binding energies (in kcal/mol) over 1 ns implicit-solvent MD simulations for taxol and the top 86 NPs within the P-gp binding pocket.[a]

| No. | Compound Name/Code | Docking Score (kcal/mol) | | | MM-GBSA Binding Energy (kcal/mol) |
|---|---|---|---|---|---|
| | | Fast | Moderate | Expensive | |
| | Taxol | –9.4 | –8.0 | –10.2 | –65.9 |
| 1 | NPC197736 | –8.8 | –10.9 | –13.5 | –90.6 |
| 2 | NPC475164 | –9.3 | –11.3 | –11.9 | –88.9 |
| 3 | NPC2313 | –9.7 | –12.1 | –12.0 | –87.4 |
| 4 | NPC104372 | –9.9 | –11.0 | –12.2 | –87.1 |
| 5 | NPC471637 | –9.7 | –11.4 | –12.2 | –84.8 |
| 6 | NPC70862 | –11.6 | –11.8 | –12.2 | –82.3 |
| 7 | NPC477344 | –10.2 | –11.1 | –11.9 | –79.6 |
| 8 | NPC477533 | –11.1 | –12.6 | –13.3 | –79.4 |
| 9 | NPC223735 | –9.9 | –11.4 | –12.6 | –78.8 |
| 10 | NPC472939 | –10.4 | –11.9 | –12.2 | –77.7 |
| 11 | NPC475655 | –9.6 | –11.1 | –12.1 | –77.6 |
| 12 | NPC472936 | –10.6 | –11.5 | –12.0 | –77.2 |
| 13 | NPC329943 | –11.4 | –11.8 | –12.0 | –76.9 |
| 14 | NPC208258 | –8.4 | –12.1 | –13.1 | –75.9 |
| 15 | NPC470582 | –10.3 | –10.5 | –12.1 | –74.7 |
| 16 | NPC276231 | –15.1 | –15.9 | –16.4 | –74.2 |
| 17 | NPC313421 | –10.5 | –12.1 | –12.1 | –72.4 |
| 18 | NPC123859 | –10.1 | –12.1 | –12.5 | –72.3 |
| 19 | NPC471860 | –11.9 | –12.1 | –12.5 | –72.2 |
| 20 | NPC149962 | –10.8 | –10.8 | –12.1 | –71.3 |
| 21 | NPC471850 | –10.7 | –12.0 | –12.6 | –70.9 |
| 22 | NPC474581 | –10.6 | –11.6 | –11.9 | –70.8 |
| 23 | NPC472437 | –13.5 | –12.9 | –14.5 | –68.1 |
| 24 | NPC231271 | –12.4 | –12.6 | –12.9 | –66.3 |
| 25 | NPC472937 | –10.3 | –11.4 | –12.1 | –66.3 |
| 26 | NPC74086 | –10.5 | –10.8 | –12.5 | –66.3 |
| 27 | NPC471583 | –11.8 | –11.8 | –11.8 | –65.7 |
| 28 | NPC471858 | –11.5 | –11.5 | –11.9 | –65.6 |
| 29 | NPC177362 | –12.0 | –12.1 | –12.2 | –64.9 |
| 30 | NPC202104 | –12.6 | –13.5 | –13.2 | –64.6 |
| 31 | NPC470601 | –9.6 | –13.0 | –14.9 | –64.5 |
| 32 | NPC65118 | –12.2 | –12.7 | –12.6 | –64.5 |
| 33 | NPC471859 | –12.1 | –12.3 | –12.3 | –63.6 |
| 34 | NPC472934 | –10.7 | –11.3 | –11.9 | –63.4 |
| 35 | NPC327962 | –12.2 | –12.7 | –12.4 | –62.7 |
| 36 | NPC477531 | –11.0 | –12.1 | –12.1 | –62.5 |
| 37 | NPC322800 | –11.2 | –11.8 | –11.8 | –62.5 |
| 38 | NPC313179 | –10.9 | –10.4 | –12.2 | –61.6 |
| 39 | NPC78159 | –11.3 | –11.0 | –11.9 | –60.0 |
| 40 | NPC473547 | –11.9 | –11.9 | –11.9 | –59.7 |
| 41 | NPC47905 | –10.8 | –12.2 | –11.9 | –58.6 |
| 42 | NPC69383 | –10.9 | –11.5 | –12.3 | –58.3 |
| 43 | NPC239990 | –11.9 | –12.3 | –12.2 | –58.3 |
| 44 | NPC231712 | –9.3 | –12.1 | –12.3 | –58.3 |
| 45 | NPC81137 | –8.8 | –11.9 | –12.0 | –58.3 |
| 46 | NPC242269 | –13.2 | –14.1 | –14.2 | –58.0 |
| 47 | NPC469671 | –10.3 | –11.8 | –12.1 | –57.7 |
| 48 | NPC65034 | –11.9 | –12.1 | –12.2 | –57.6 |
| 49 | NPC4638 | –12.2 | –11.5 | –12.2 | –57.5 |
| 50 | NPC477874 | –11.7 | –11.8 | –12.0 | –57.3 |
| 51 | NPC470426 | –12.8 | –12.8 | –12.8 | –57.3 |
| 52 | NPC202428 | –11.2 | –11.8 | –11.9 | –57.2 |
| 53 | NPC162440 | –12.2 | –12.2 | –12.2 | –56.1 |
| 54 | NPC126676 | –11.8 | –11.6 | –12.5 | –55.7 |
| 55 | NPC133209 | –10.8 | –11.5 | –12.3 | –55.4 |

**Table S2.** *Continued*.

| No. | Compound Name/Code | Docking Score (kcal/mol) | | | MM-GBSA Binding Energy (kcal/mol) |
|---|---|---|---|---|---|
| | | Standard | Moderate | Expensive | |
| 56 | NPC266545 | –11.0 | –10.9 | –12.1 | –55.2 |
| 57 | NPC473403 | –10.5 | –12.3 | –12.4 | –55.1 |
| 58 | NPC475493 | –13.4 | –13.7 | –13.8 | –54.1 |
| 59 | NPC469447 | –12.1 | –12.4 | –12.3 | –54.0 |
| 60 | NPC96605 | –8.5 | –10.6 | –11.9 | –53.9 |
| 61 | NPC224528 | –12.3 | –12.6 | –12.8 | –53.7 |
| 62 | NPC105942 | –11.5 | –12.0 | –12.1 | –53.4 |
| 63 | NPC472395 | –11.9 | –12.1 | –12.9 | –53.1 |
| 64 | NPC471863 | –12.1 | –11.9 | –11.9 | –52.6 |
| 65 | NPC472236 | –11.2 | –12.4 | –12.4 | –52.4 |
| 66 | NPC183736 | –10.9 | –12.3 | –12.5 | –52.3 |
| 67 | NPC472371 | –12.4 | –12.6 | –12.1 | –52.1 |
| 68 | NPC473507 | –10.1 | –11.5 | –12.9 | –51.7 |
| 69 | NPC270958 | –10.2 | –11.4 | –12.0 | –51.4 |
| 70 | NPC475258 | –12.7 | –12.7 | –12.7 | –50.7 |
| 71 | NPC475570 | –11.7 | –11.8 | –11.8 | –50.3 |
| 72 | NPC103250 | –12.0 | –12.1 | –12.1 | –50.1 |
| 73 | NPC307205 | –12.4 | –12.4 | –12.4 | –50.0 |
| 74 | NPC473612 | –11.7 | –12.0 | –12.0 | –49.9 |
| 75 | NPC36754 | –12.0 | –12.0 | –12.0 | –49.3 |
| 76 | NPC473720 | –11.8 | –11.9 | –11.9 | –48.6 |
| 77 | NPC143173 | –12.0 | –12.0 | –12.0 | –48.1 |
| 78 | NPC250835 | –12.2 | –12.2 | –12.2 | –47.9 |
| 79 | NPC6702 | –12.0 | –12.5 | –12.7 | –47.1 |
| 80 | NPC94531 | –11.0 | –11.5 | –11.8 | –46.9 |
| 81 | NPC475520 | –11.9 | –11.8 | –11.9 | –46.8 |
| 82 | NPC477207 | –11.9 | –12.5 | –12.6 | –46.5 |
| 83 | NPC208011 | –11.0 | –10.9 | –12.1 | –45.2 |
| 84 | NPC127197 | –12.0 | –12.0 | –12.0 | –43.0 |
| 85 | NPC9856 | –11.8 | –11.8 | –11.8 | –40.4 |
| 86 | NPC470602 | –10.9 | –13.0 | –14.7 | –22.6 |

[a]Data ranked based on the MM-GBSA binding energy over the 1 ns implicit MD simulations.
